# Shade Effects on Peanut Yield Associate with Physiological and Expressional Regulation on Photosynthesis and Sucrose Metabolism

**DOI:** 10.3390/ijms21155284

**Published:** 2020-07-25

**Authors:** Tingting Chen, Huajian Zhang, Ruier Zeng, Xinyue Wang, Luping Huang, Leidi Wang, Xuewen Wang, Lei Zhang

**Affiliations:** 1 Guangdong Key Laboratory of Plant Molecular Breeding, State Key Laboratory for Conservation and Utilization of Subtropical Agro-Bioresources, College of Agriculture, South China Agricultural University, Guangzhou 510642, China; chentingting@scau.edu.cn (T.C.); 15099976174@163.com (H.Z.); ruierzeng@126.com (R.Z.); wangxinyuescau@163.com (X.W.); lupinghuang2019@163.com (L.H.); wangld@mail.iap.ac.cn (L.W.); 2 Department of Genetics, University of Georgia, Athens, GA 30602, USA

**Keywords:** *Arachis hypogeae* L., shade, transcriptome, yield, metabolism, photosynthesis

## Abstract

Intercropping improves land utilization with more crops grown together; however, shorter crops in intercropping experience stress, being shaded by the taller crops. Systematic changes in phenotype, physiology, yield, and gene regulation under shade stress in peanut are largely unknown, although shade responses have been well analyzed in model plants. We exposed peanut plants to simulated 40% and 80% shade for 15 and 30 days at the seedling stage, flowering stage, and both stages. Shade caused the increased elongation growth of the main stem, internode, and leaf, and elongation was positively associated with auxin levels. Shade stress reduced peanut yield. Further comparative RNA-seq analyses revealed expressional changes in many metabolism pathways and common core sets of expressional regulations in all shade treatments. Expressional downregulation of most genes for light-harvesting and photosynthesis agreed with the observed decreased parameters of photosynthesis processes. Other major regulations included expressional downregulation of most core genes in the sucrose and starch metabolism, and growth-promoting genes in plant hormone signal pathways. Together, the results advance our understanding of physiological and molecular regulation in shade avoidance in peanut, which could guide the breeding designing in the intercropping system.

## 1. Introduction

With increasing demand for food and the decreasing availability of arable land [1,2], intercropping, a practice where multiple crop species are grown together, is gaining in popularity as a sustainable practice for low-input or resource-limited agricultural systems [3]. Several intercropping systems, such as maize/soybeans [4,5,6], maize/peanut [7], cotton/peanut [8], and sugarcane/soybean [9], have been developed and have proven to be efficient. High and low tiers of intercropped plants may benefit from each other, especially for leguminous crops and others [3]. However, interspecific competition for light and fertilizer is the main factor constraining plant growth and yield in the intercropping systems [10].

Shading emerges as a result of high-density planting and intercropping, which reduces light intensity and changes the light quality to a low ratio of red light to far-red light [11,12]. A low-height crop is always affected by a tall crop in the intercropping practice, receiving a reduced amount of sunlight. It is known that plants will try to escape shade, after perceiving the change of light signal in shade caused by plant proximity, through shade avoidance. Shade avoidance syndrome (SAS) includes elongation growth, accelerated flowering, early seed production, and reduced yield [13]. Photoreceptor phytochromes perceive a change in the ratio of red light to far-red light under shade, and photoreceptor cryptochromes sense the change of light intensity to control adaptive developmental strategies [14,15,16,17]. The signal then is transduced and cascaded via PHYTOCHROME INTERACTING FACTORS (PIFS), circadian clock basic helix–loop–helix protein PIL1, circadian clock protein TOC1, and other transcription factors in DELLA families to trigger changes of gene expression and induce a series of SAS responses [11,12,14,18,19]. Many genes were involved in light, hormone, and stress responses in early responses to low ratio of red and far-red light of shade [20,21,22,23]. Comparative RNA-Seq analyses in conifers with different syndromes of shade avoidance and tolerance show that main transcriptional regulations are involved in hormone signaling and pigment biosynthesis [24]. Crosstalk of shade avoidance has been found between transcription factors, hormones, and circadian clock etc. in plants [14]. Growth elongation of the stem and petioles is to avoid shading at the cost of assimilated resources, which eventually reduces the crop yield [25,26]. Under intercropping, shading occurs for the low-tier plant where the photosynthetically active radiation (PAR) decreases [27]. The SAS is balanced plastic responses from interactions of both PAR and the low ratio of red to far-red light [28,29]. Most knowledge of shade studies has been gained from model plants e.g. *Arabidopsis*, instead of crops.

Peanut *Arachis hypogaea* L. (Fabaceae), also known as groundnut, is an important oil and protein crop of South American origin and is nowadays cultivated worldwide [30]. The cultivated peanut is a tetraploid that was derived from an ancient hybridization of two diploid peanut ancestors. The genome sequences of ancestral diploid peanuts with ~1.2 Gb genome A in *A. duranensis* or B in *A. ipaensis* [31,32], and genome sequences of three cultivars of *A. hypogaea* with genome AABB of 2.6 Gb are made available now [33,34,35], which has facilitated the exploration of molecular mechanisms of physiological processes in peanut, including SAS. The SAS effect is of high practical importance due to the decreasing intensity of sunlight in China and other countries as a result of pollution [36].

In this study, we simulated 40% and 80% shade to treat peanut plants at the seedling stage, flowering stage, and combined both stages (CS) for two periods (15 days and 30 days, respectively) while kept the control plants under natural sunlight. To understand the shade effects on growth, biomass, and seed yield, we first examined the physiological and hormonal changes. Then, to understand the gene regulatory mechanisms of peanut plant responses to shade, we generated and compared the genome-scale transcriptome profiles for shaded and control leaves. We identified common expressional regulation under different shade schemes involved in photosynthesis pathways, starch and sucrose metabolism, and hormonal signal pathways. We found that shade stresses induced expressional reduction of genes in light-harvesting complex and were associated with altered expression of genes in photosynthesis systems, which resulted in low physiological photosynthesis. Shade stress also caused the common downregulation of key genes in starch and sucrose metabolism and hormonal signal pathways. Shading degree, duration, and developmental stages were also compared to reveal the difference of effects. Our results demonstrated the association of gene expressional regulation with strong SAS in peanut plants under shade stress. This study provided clues to physiological changes and gene regulatory mechanisms of shade avoidance syndrome in peanut plants, which advances our understanding of shade avoidance in the peanut crop. Thus, our results may guide the design of intercropping and breeding by tuning the associated gene expression.

## 2. Results

### 2.1. Effects of Shade Stress on Size of Peanut Plants and Association with Auxin

**Figure 1:**
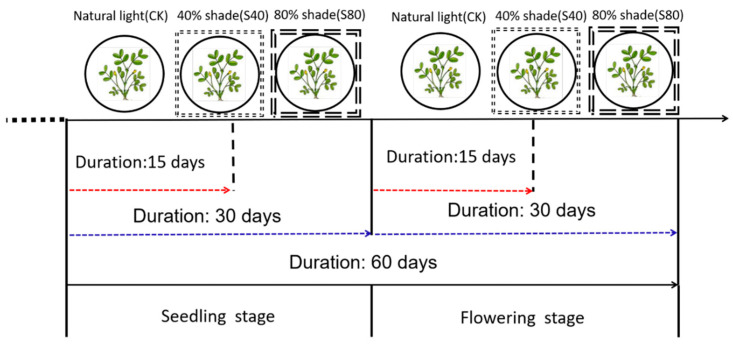
Shading treatment scheme in peanut plants.

We treated peanut plants with 40% and 80% shade for 15 or 30 days at the seedling stage, flowering stage, and CS stage while kept the control under natural sunlight (Figure 1). The length of the main stem and the longest internode, the third internode counting from bottom, significantly increased (*p* < 0.05) under 40% and 80% shade compared with the control at the flowering stage and CS stage. The greatest effect on stem length was observed at the CS stage, which increased by 75% and 119% under 40% and 80% shade compared with the control, respectively. Both shade degree and duration had positive effects on the length of the main stem and internode and had negative effects on the diameter of the main stem (Table 1). The thinnest main stem was found after the shade treatments at the CS stage. There was no significant effect of the shade treatment on the number of leaves at the flowering stage; however, the effect was significant at the CS stage, which may be associated with different leaf developmental speeds across stages (Table 1).

**Figure 2:**
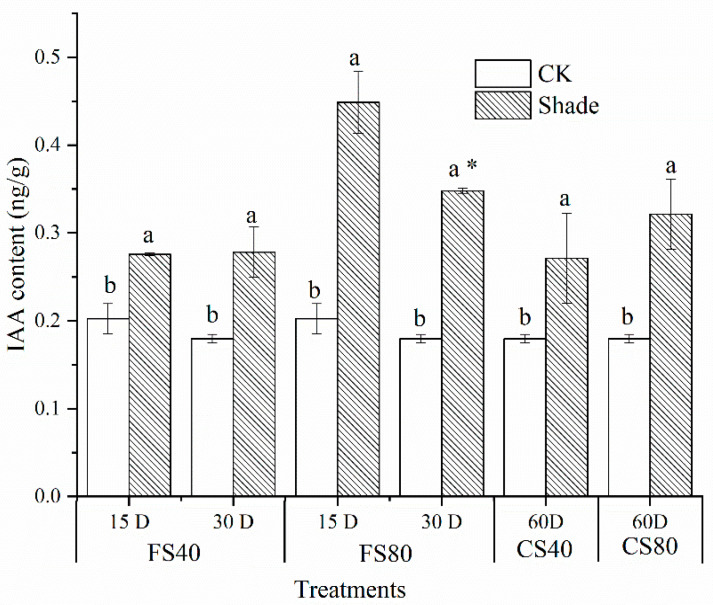
Effects of shade stress on indole-3-acetic acid contents in peanut leaves. Data represent mean ± standard error from three experimental replicates, each with three plants. The indole-3-acetic acid (IAA) was measured in leaves on the main stem after 40% and 80% shading for 15 days, 30 days at the flowering stage (FS), and shading from the seeding stage for 30 days to the flowering stage for another 30 days (CS). The IAA content was calculated as ng per gram fresh leaf weight. Letters a and b after the value represent the statistically significant difference (*p* < 0.05) between any two conditions within a stage as determined by the Least Significant Difference test. * represents a significant difference (*p* < 0.05) between different treating durations of 15 days and 30 days. D for days, CK for control under natural sunlight at the same time point.

To test whether the increase in length was associated with auxin, we tested levels of indole-3-acetic acid (IAA) in leaves. Levels of IAA were increased in all shade stress treated samples compared with those in corresponding control at the same time point under natural light (Figure 2). 

### 2.2. Effects of Shade Stress on Yield Components 

Peanut seeds were produced under all shade treatments and in the control with natural sunlight of 131000 lux intensity. The peanut yield components of total pods per plants, 100-pod weight, and 100-kernel weight were measured after shade treatments and control (Table 2). At all three developmental stages, the pod number per plant, 100-pod weight, and 100-kernel weight were significantly decreased (*p* < 0.05) under all shade treatments compared with those in corresponding natural-light control. There was no difference in total pods per plant between 40% and 80% shade treatments under the same duration of shade. The 40% shade for 30 days caused a greater reduction than that for 15 days. Regarding the 100-pod weight and 100-kernel weight, the 30-day shade caused a greater decrease than 15-day shade under the same shade level. Under shade, lower 100-pod weight and 100-kernel weight were found at the flowering stage than at the seedling stage. The shading at the CS stage led to the lowest 100-pod weight and 100-kernel weight (Table 2).

### 2.3. Transcriptome Profiles and Expressional Regulation Responsive to Shade

**Figure 3:**
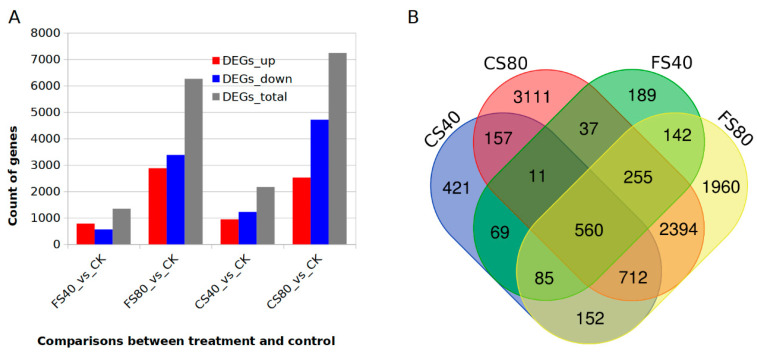
Comparison of DEGs induced by different shade stresses in peanut plants. (**A**). The number of up-, down- and total- regulated differentially expressed genes (DEGs) induced by different shading treatments compared with control (CK) under natural sunlight in peanut plants. (**B**). Venn graph showing the common and specific DEGs across samples after shade treatments. FS40, FS80, CS40, and CS80 represent shade treatments of 40% shade at flowering stage for 30 days, 80% shade at flowering stage for 30 days, 40% shade from seeding stage for 30 days to flowering stage for another 30 days, 80% shade from seeding stage for 30 days to flowering stage for another 30 days, respectively. Three experiments were conducted, each with three plants.

To understand the gene regulation induced by different shade treatments, we generated the transcriptome profiles using a deep RNA-Seq technology for control CK and shade treatments FS40 for 30 days, FS80 for 30 days, CS40 for 60 days, and CS80 for 60 days. Around 8 Gb paired-end Illumina reads were independently generated for each sample in triplicate experiments. After cleaning and mapping reads to peanut reference genome of cultivar Tifrunner [34], the transcripts were constructed with HiSat2 and Stringtie [37], and differentially expressed genes (DEGs) were identified with DESeq2 [38] using previously described parameters [39]. We detected 71,324 expressed genes in all samples. Relative to the transcript abundance in control, we identified 1348, 6260, 2167, and 7237 DEGs in samples with treatments FS40, FS80, CS40, and CS80, respectively (Figure 3A, Data S1, Data S2, Data S3, Data S4,). The 40% shade induced fewer DEGs than 80% shade at both the flowering stage and CS stage, suggesting that a severe shade led to more changes in gene expression in peanut plants. The 80% shade treatment caused more down-regulated DEGs than up-regulated DEGs at both stages of FS and CS. More DEGs were identified in the CS stage than FS, meaning a longer shading caused more expressional changes (Figure 3A). Comparisons of DEGs across samples revealed that 560 genes were shared by all shade treatments, indicating a core set of regulation in response to shade (Figure 3B).

### 2.4. Function of Induced DEGs and Affected Pathways by Shade Stress

**Figure 4:**
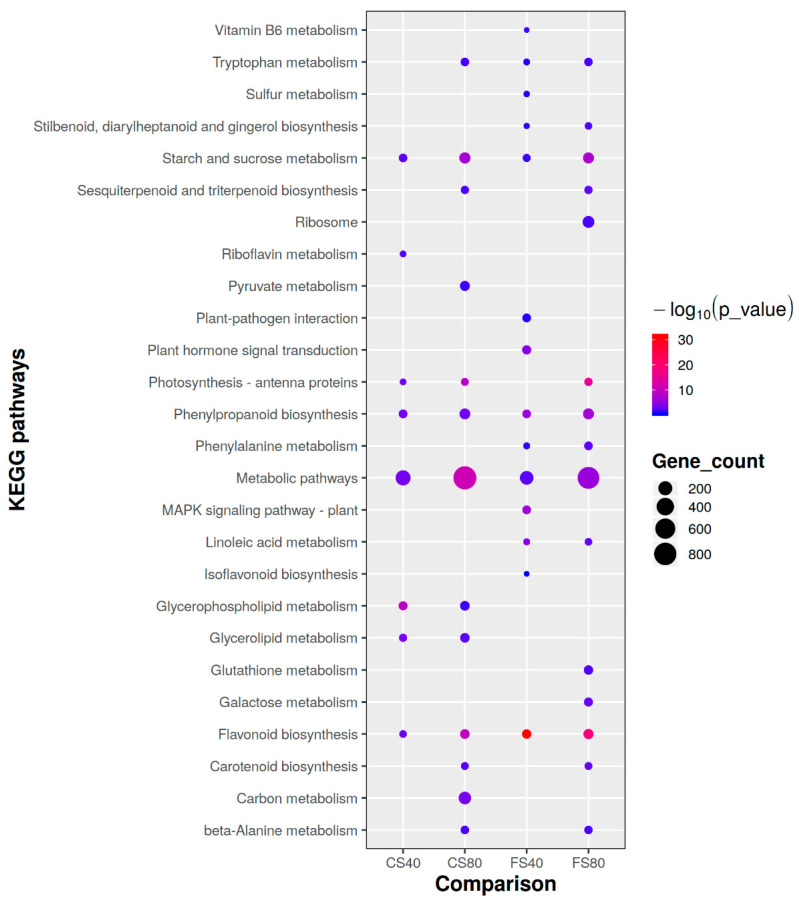
Comparison of enriched metabolism pathways of DEGs induced by shade stress in peanut plants. Only the significantly affected (hypergeometric test *p* < 0.05) involved by shade induced DEGs were plotted here. The pathways information is from the database KEGG (https://www.genome.jp/kegg/). FS40, FS80, CS40, and CS80 represent DEGs, relative to control under natural sunlight, mined from shade treatments of 40% shade at flowering stage for 30 days, 80% shade at flowering stage for 30 days, 40% shade from seeding stage for 30 days to flowering stage for another 30 days, 80% shade from seeding stage for 30 days to flowering stage for another 30 days, respectively. Three experiments were conducted, each with three plants.

To further understand DEGs’ roles in shade responses, we first conducted an enrichment analysis of their Gene Ontology (GOs) and found that 76 GOs were enriched, including carbohydrate metabolic process, reproductive process, cell wall organization biogenesis, photosynthesis, signal transduction, and transportation. Further metabolism pathway enrichment analysis against the database KEGG (https://www.genome.jp/kegg/) identified 26 enriched pathways (Figure 4). Among those, Tryptophan metabolism for indolic acid biosynthesis, Starch and sucrose metabolism, Photosynthesis-antenna proteins, Phenylpropanoid biosynthesis, Flavonoid biosynthesis, and general metabolic pathways were significantly affected (hypergeometric test, *p* < 0.05) in at least three of four shade treatments (Figure 4). The 80% shade affected more pathways than the 40% shade. These results suggested that large effects of shade on expressional regulation were involved in photosynthesis light capture, assimilation of major carbohydrates, defensive metabolites, and hormone biosynthesis.

### 2.5. Affected Metabolism Pathways Involved by Common DEGs

**Figure 5:**
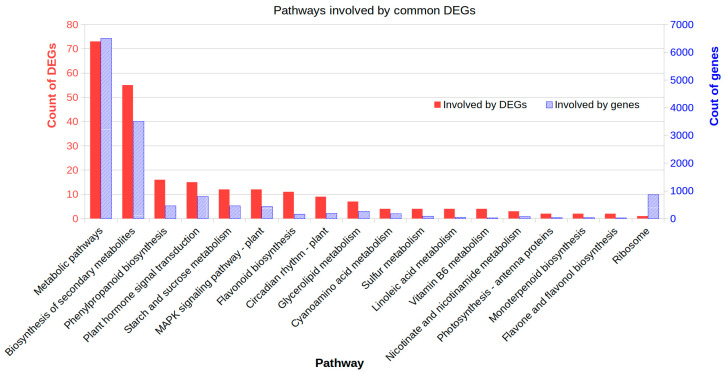
Affected metabolism pathways enriched by common differentially expressed genes (DEGs) induced in all shade treatments. The common DEGs shared by all shade treatments were mapped to the KEGG database (https://www.genome.jp/kegg) and pathway names were extracted. The enrichment was conducted for DEGs involved in each pathway using the genome-scale expressed genes as the background with a hypergeometric test. The plot shows only the significantly enriched pathways (*p* < 0.05).

We further analyzed the affected metabolism pathways of the 560 commonly induced DEGs across all four shade treatments (Data S5). These common DEGs were involved in 64 pathways in the database KEGG. Of those, 18 pathways were significantly affected (Hypergeometric test *p* < 0.05) (Table S1). These pathways were Biosynthesis of secondary metabolites, Phenylpropanoid biosynthesis, Plant hormone signal transduction, Starch and sucrose metabolism, MAPK signaling pathway, Flavonoid biosynthesis, Circadian rhythm, Glycerolipid metabolism, Cyanoamino acid metabolism, Sulfur metabolism, Linoleic acid metabolism, Vitamin B6 metabolism, Nicotinate and nicotinamide metabolism, Photosynthesis - antenna proteins, Monoterpenoid biosynthesis, Flavone and flavonol biosynthesis, and Ribosome. The changes of these pathways should represent common responses to shade no matter at seedling stages, flowering stage, and both stages (Figure 5).

### 2.6. Regulation of DEGs and Association with Physiological Photosynthesis

**Figure 6:**
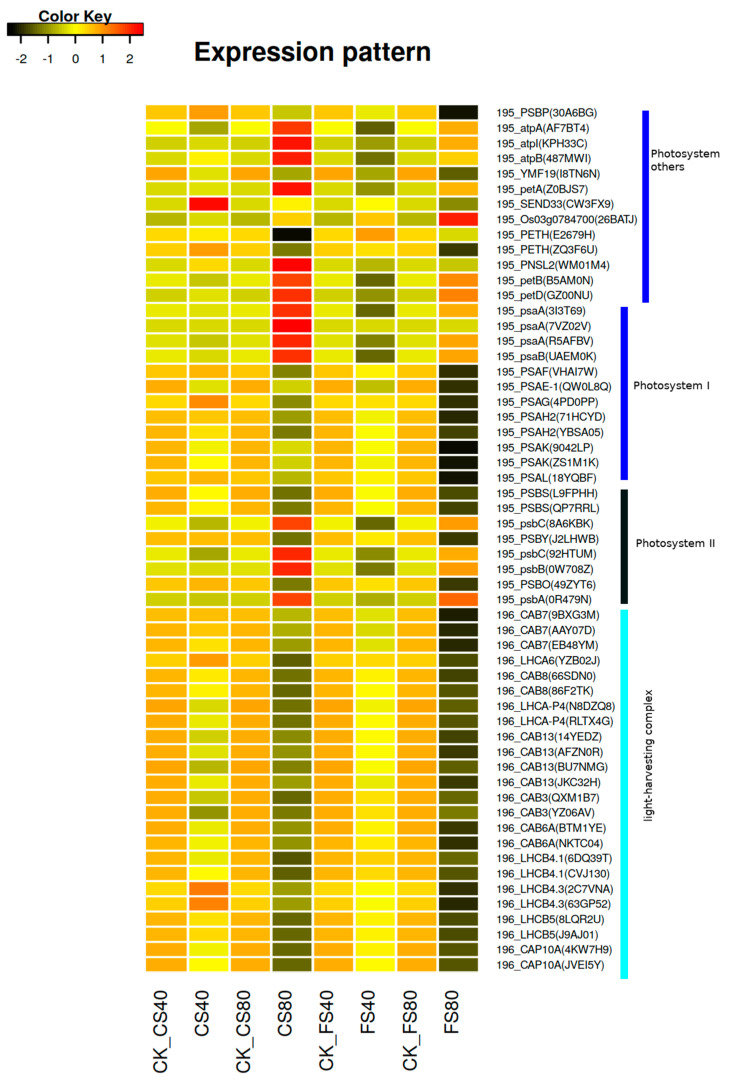
Expression patterns of DEGs induced by shade stress in photosynthesis pathways. The heatmap showing expression levels of differentially expressed genes (DEGs) induced by shade treatments in leaves of peanut plants. The expression level of each gene was calculated as fragment per kilobase per million reads (FPKM) from RNA-Seq data. The color represents the z-score after the transformation of log2(mean FPKM). Each row represents the levels of a DEG and the name of the DEG was listed on the right side of each row. These DEGs involved in the photosynthesis pathway were tagged with a prefix 195 (KEGG map 00195 at https://www.genome.jp/kegg/pathway/map/map00195.html) and photosynthesis antenna pathway with a prefix 196 (https://www.genome.jp/kegg/pathway/map/map00196.html). The abbreviation of DEGs listed in Table 3 and Table 4 were extracted from KEGG (www.genome.jp/kegg/) based on the best match to the homologous reference genes in the database KEGG; and corresponding gene id in the peanut genome assembly was listed in parenthesis in the heatmap. FS40, FS80, CS40, and CS80 represent shade treatments of 40% shade at the flowering stage for 30 days, 80% shade at the flowering stage for 30 days, 40% shade from the seeding stage for 30 days to the flowering stage for another 30 days, 80% shade from the seeding stage for 30 days to the flowering stage for another 30 days, respectively. Three experiments were conducted, each with three plants. CK represents control under natural light.

Since shade induced DEGs were enriched in the photosynthesis antenna pathway, we examined the detailed functions and expressional regulation of the DEGs in this pathway. Firstly, DEGs in the photosynthesis antenna pathway were identified in each shade treatment compared with control under natural light; and then annotated against the pathway database KEGG via the tool KAAS [39,40]. In total, 24 DEGs were identified in the photosynthesis antenna pathway (KEGG map id 00196, https://www.genome.jp/kegg/pathway/map/map00196.html), and annotated to encode proteins of two light-harvesting chlorophyll complexes (Table 3). Comparisons of expression levels revealed downregulation of those DEGs at shade treatments except that LHCA6 (gene id YZB02J), LHCB4.3 (gene id 2C7VNA) and LHCB4.3 (63GP52) showed an increase at shade treatment CS40 compared with control (Figure 6, Table S2). We inferred that the downregulation of light-harvesting complex encoding genes could reduce the efficiency of photosynthesis. Therefore, we further investigated DEGs in the photosynthesis pathway (KEGG map id 00195, https://www.genome.jp/kegg/pathway/map/map00195.html). We found a positive association between the abundance of light-harvesting complex encoding genes and the abundance of DEGs in the photosynthesis pathway, where 80% shade treatments induced much lower expression than 40% shade treatments (Figure 6). However, we found treatment CS80 also induced an increase of some DEGs while CS40 treatment decreased the expression of most DEGs. CS80 induced an increased expression of four DEGs of *psbC* (8A6KBK, 92HTUM), *psbB* (0W708Z), *psbA* (0R479N) in photosystem II, four DEGs of *pasA* (3I3T69, 7VZ02V, R5AFBV), *psaB* (UAEM0K) photosystem I, seven DEGs of *petD* (GZ00NU), *petB* (B5AM0N), *PNSL2* (WM01M4), *petA* (Z0BJS7), *atpB* (487MWI), *atpI* (KPH33C) and *atpA* (AF7BT4) in other systems of photosynthesis. The expression of *pasA* (7VZ02V) was zero in control and it was only activated after treatment CS80 although at a very low level with the FPKM of 0.3, which may suggest a specific regulation after a very long shade treatment. At the flowering stage, the treatment FS80 induced a greater expressional decrease of most DEGs than the treatment FS40 did. Similarly, the expression levels of some DEGs were increased with FS80, but the increase was much lower than that with CS80 treatment. Those increased DEGs were shared between FS80 and CS80, meaning similar regulatory patterns under shade stresses (Figure 6).

**Figure 7:**
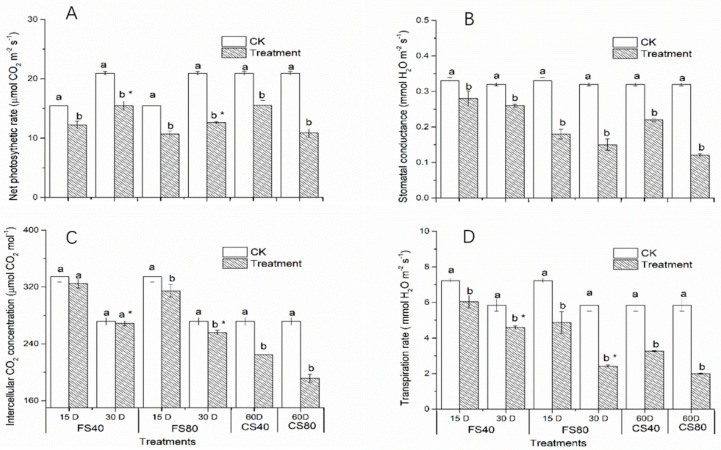
Effects of shade stress on photosynthesis parameters in peanut leaves. (**A**). Changes in net photosynthetic rate, (**B**). Changes in stomatal conductance, (**C**). Changes in intercellular CO_2_ concentration, (**D**). Changes in transpiration rate. Data represent mean ± standard error. The measurement was conducted on leaves on the main stem after a treatment. Letters a and b after the value represent the statistically significant difference (*p* < 0.05) between any two conditions within a stage as determined by the Least Significant Difference test. * represents a significant difference at *p* < 0.05 between different treating durations of 15 days and 30 days. FS40, FS80, CS40, and CS80 represent shade treatments of 40% shade at the flowering stage for 30 days, 80% shade at the flowering stage for 30 days, 40% shade from the seeding stage for 30 days to the flowering stage for another 30 days, 80% shade from the seeding stage for 30 days to the flowering stage for another 30 days, respectively. Three experiments were conducted, each with three plants. CK represents control under natural light.

To further validate the effects on photosynthesis, we checked the physiological parameters of photosynthesis. Results revealed that all shade treatments induced a significant decrease (*p* < 0.05) of net photosynthetic rate (Pn) compared with corresponding control under natural sunlight at the flowering stage and CS stage (Figure 7A), suggesting that low expression of DEGs in photosynthesis pathway is associated with low physiological photosynthesis process. The 80% shade stress led to lower Pn than 40% shade under the same duration. The shade treatments induced similar changes in the stomatal conductance (Figure 7B). The intercellular CO_2_ concentration (Ci) was not significantly affected by the 40% shade stress for either 15 or 30 days at the flowering stage, while the 80% shade stress for 30 days significantly decreased Ci at the flowering stage (Figure 7C). Both 40% and 80% shade stresses, of which plants were exposed to for 15 days and 30 days, led to a significant decrease in transpiration rate at the flowering stage and stage CS (Figure 7D). Together, these results suggest that altered expression of DEGs in photosynthesis pathways, observed under shade stress, led to a decrease in photosynthetic activity.

### 2.7. Regulation of DEGs in Starch and Sucrose Metabolism and Association with Biomass

**Figure 8:**
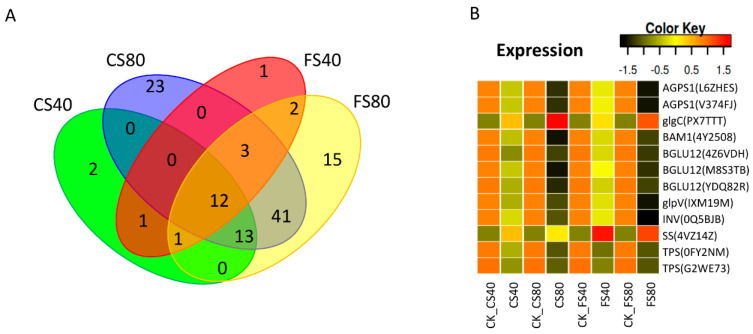
Shade induced common DEGs in starch and sucrose metabolism and their expression patterns. (**A**). Venn graph showing the common and specific DEGs induced by shade stresses. (**B**). The expression levels of DEGs in starch and sucrose metabolism. The expression level was calculated as fragment per kilobase per million reads (FPKM) from RNA-Seq data. The color in heatmap represents the z-score after the transformation of log2(mean FPKM). Each row in the heatmap represents the levels of a DEG under different conditions. DEG’s name and gene ID in parenthesis were listed on the right side of the heatmap. The abbreviation of each DEG is given in Table 5. FS40, FS80, CS40, and CS80 represent shade treatments of 40% shade at the flowering stage for 30 days, 80% shade at the flowering stage for 30 days, 40% shade from the seeding stage for 30 days to the flowering stage for another 30 days, 80% shade from the seeding stage for 30 days to the flowering stage for another 30 days, respectively. Three experiments were conducted, each with three plants. CK represents the control under natural sunlight.

We further investigated the regulation of DEGs enriched in the pathway of starch and sucrose metabolism. In total, 113 DEGs were induced by all shade stress treatments (Table S3). Of those, more DEGs were identified at 80% shade stress than at 40% shade stress. Most DEGs were downregulated under shade stress compared with those in corresponding control with natural light. Comparisons of DEGs showed that 12 DEGs were shared by all shade stress treatments at the flowering and CS stages, which should be the key DEGs for the starch and sucrose metabolism (Figure 8A). Further annotation of these shared DEGs reveals that they encode enzymes mostly for catalyzing the intermediates for sucrose and starch synthesis or degrading, e.g. glycodidase AGPS1 (Table 5). Comparisons of expression levels identified that 10 out of 12 DEGs in sucrose and starch synthesis were downregulated in response to shade stress. The common DEGs’ expression levels were much lower in the 80% shade treatment than those in the 40% shade treatment (Figure 8B). DEG encoding sucrose synthase 4 (SS or SUS), which catalyzes both sucrose synthesis and hydrolysis. The sucrose hydrolysis in leaves occurs via activation of invertase (INV) in the vacuole [41,42,43]. Here, SS was upregulated and INV was downregulated under shade compared with that in control under natural light (Figure 8B), indicating that sucrose hydrolysis in the leaf was inhibited under shade. Another DEG, encoding glucose-1-phosphate adenylyltransferase (glgC) for the synthesis of ADP-glucose as an exclusive donor of glucose moiety for starch synthesis was upregulated while the other two DEGs called *AGPS1* encoding the same enzyme were downregulated. Enzymes of beta amylase (BAM1) and starch phosphorylases (three forms BGLU12 and glpV), which catalyze starch degradation, were downregulated. Therefore, the altered expression of these DEGs in this pathway may indicate a reduced hydrolysis of sucrose and starch in chloroplast under shade stress. Besides, six DEGs including *0FY2NM*, *IEIY3V*, *2A3E3X*, *P5P8C7*, *PH1SAB,* and *W2MXXV* were silenced (0 FPKM) with 80% shade compared with those in natural control, in which these DEGs were lowly expressed. DEGs of *NH7NM* and *477F6P* were found to be not expressed in natural-light control but were activated to a lowly expressed level in shade treated samples.

Sucrose and starch metabolism provide many intermediates for carbohydrates as the precursor of the major components of biomass. Therefore, we further checked dry biomass of main stem, leaves, and roots, and revealed that the shade stress reduced the biomass. A significant reduction (*p* < 0.05) was found in the 80% shade treatment and 40% shade treatment for 30 days. The biomass reduction in root and stem were not significant under 40% shade stress for 15 days; however, the reduction in leaf was significant, which suggested that the leaf, where the carbon assimilation is initially processed, was earlier affected than stem and root by shade stress (Table 6), and the significant reduction was time-dependent. Together, shade stress led to a reduction in biomass and biased resources reallocation under long-term shade stresses compared with those under natural light.

### 2.8. Regulation of DEGs in Hormone Signaling 

**Figure 9:**
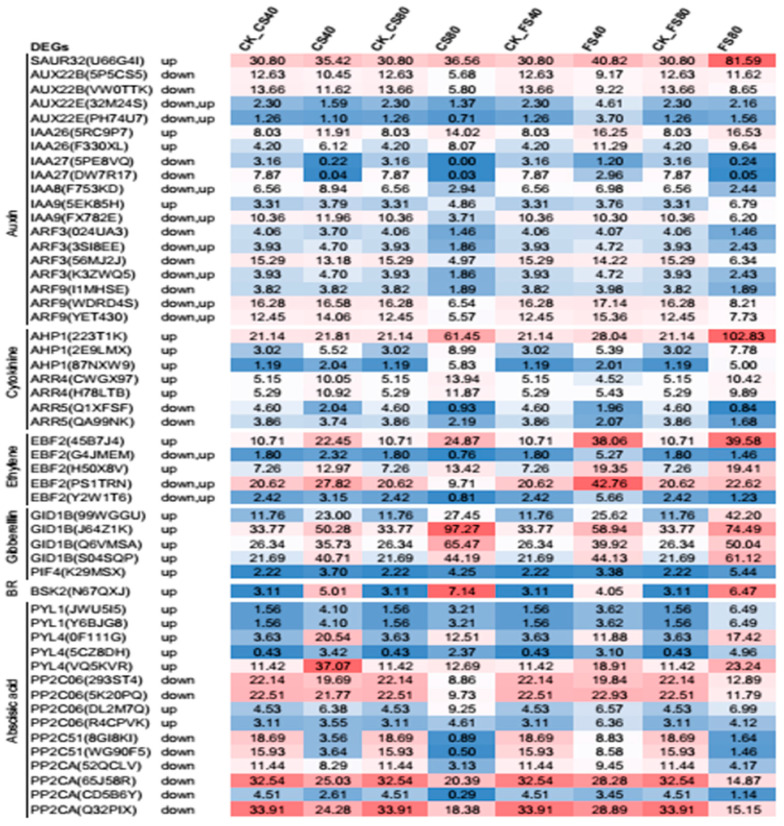
Gene expressional regulation in multiple hormone signal pathways. Abbreviates for DEG encoding proteins: SAUR, small auxin up RNAs like protein, AUX, auxin influx carrier; IAA, auxin-responsive protein IAA; ARF, auxin response factor; AHP, histidine-containing phosphotransferase; A-ARR, two-component response regulator ARR-A family; PYL, abscisic acid receptor PYL family; PP2C, protein phosphatase 2C; EBF, ethylene EIN3-binding F-box protein 2; GID1, gibberellin receptor GID1; PIF, phytochrome interacting factor 4; BR, brassinosteroids, BSK, brassinosteroid signaling kinase; Expression data represent the mean value of fragment per kilo base pair per million reads from three replicates, each with three biological plants at each treatment and corresponding control.

The increased IAA level in peanut leaves could crosstalk to other plant hormones to regulate shade avoidance [44]. We further investigated the DEG regulation in multiple hormone signal transduction in this study against the database KEGG. Our results showed that DEGs in six hormone signaling pathways were regulated under shade (Figure 9). DEG encoding Small Auxin Up RNAs-like protein (*SAUR*, gene id U66G4I) in the auxin signal pathway was highly expressed and upregulated in all shade treatments relative to that in natural light, which agreed with known knowledge [44,45]; Shade induced a greater increase of *SAUR* at the flowering stage than CS stage. The 80% shade induced a greater increase of *SAUR* than the 40% shade. Multiple copies of DEGs encoding auxin influx carrier (AUX), auxin-responsive protein (IAA), and auxin response factor (ARF) in the auxin signal pathway were found to be downregulated. However, few of the DEGs in this pathway were up regulated depending on the shade treatment stage and the degree of shade. In the gibberellin signal pathway, four copies of DEGs encoding gibberellin receptor 1 (GID1) and one DEG encoding phytochrome interacting factor 4 (PIF4) were upregulated in response to all shade treatments, and the shading at the CS stage induced a greater increase in *GID* than at the flowering stage. One DEG encoding brassinosteroid signaling kinase (BSK2) was upregulated in all shade treatments and the 80% shade induced a greater increase than the 40% shade treatment. In the ethylene signal pathway, five DEGs encoding ethylene EIN3-binding F-box protein 2 (EBF2) were upregulated in most shade treatments. In the cytokinine signal pathway, DEGs encoding histidine-containing phosphotransferase (AHP) and two-component response regulator ARR-A family (A-ARR4) were upregulated by shade treatments, which is known to interact with shade signal receptor phytochrome B, but the expression of *ARR5* was down-regulated, which may be a feedback regulation on more stable ARR5 upon increased cytokinine [46] under shade. In the abscisic acid signal pathway, five DEGs encoding the same abscisic acid receptor PYL family (PYL) were upregulated, which suppressed downstream DEGs encoding protein phosphatase 2C type A (PP2C) and most of the other PP2C types as expected [47] (Figure 9).

## 3. Discussion

Shade avoidance in plants is an adaptive response to avoid shade stress. To date, many studies show that shade avoidance is induced by light regimes with a reduced light intensity or PAR, and/or low ratio of red to far-red light [14,15,48]. In intercropping systems such as maize/peanut [7] and cotton/peanut [8], the peanut plant is shade stressed which reduced the yield of peanut. For intercropping with peanut, the existing knowledge is focused on biomass and physiological changes [8]. Here, our results revealed the systematic effects of simulated shades on phenotypic, physiological, and expressional regulation in peanut, which advances our understanding of shade regulation in intercropping. To our best knowledge, this is the first report on shade avoidance responses at the whole transcriptome scale to reveal the common core regulation of expression across shade stresses in peanut plants. Our results reveal that tuning the expressional regulation under shade could be the fundamental solution to avoid shade syndrome and to avoid yield decrease of peanut. Of course, it is of priority to further examine the expressional regulation in field shading in the real intercropping farming.

Shade stress changes the growth, photosynthesis, assimilation, and allocation of resources. As was observed in our study, decreased biomass and increased elongation growth are common characteristics of shade avoidance in plants [14,15,48]. Elongation growth is a typical shade response to low ratio of red to far-red light [17]; the latter changes auxin synthesis, transportation, perception and signaling via free auxin levels, and expression of IAA associated transcription factors [15,44]. A study of elongation of the hypocotyl in *Arabidopsis* under shade shows that cotyledon-derived auxin is necessary to initiate hypocotyl growth [23]. In our study, elongation was associated with an increase in auxin levels in leaves, which suggests that shade induces a crosstalk within hormones [44] and the auxin promotes elongation under shade [49]. Our analysis of gene regulation in multiple plant hormone signal pathways revealed that the growth promoting hormones auxin, gibberellin, brassinosteroid, cytokinine and senescing hormones of abscisic acid and ethylene were all stimulated under shade treatment. This could be a crosstalk from a manner of hormone cascaded signaling network [44,47,50]. The regulation on the hormone DEGs activates downstream regulation to promote growth elongation under shade [45]. The gene expressional crosstalk and associated hormone levels in both leaves and stem should be investigated further under shade treatment, especially for the seedling stage, where elongation is the most significant. It was observed that under shade, reduced photosynthetic activity was the result of the reduced availability of PAR. This can be explained by low expression of light-harvesting complex genes, and low expression of photosynthesis genes in our core DEG sets in photosynthesis pathways. The association between low PAR and low photosynthetic activity could be an economic and adaptive response where low concentration light-harvesting protein is enough to capture the available light energy for photosynthesis under shade, which is to optimize the use of resources under shade [15]. Several DEGs, which may have multiple homologous genes, were upregulated in the long duration shading treatment CS80 but downregulated in other short duration shade treatments, which could be a plant response to extreme stress induced by a deep and prolonged shade (i.e., too little light to maintain necessary photosynthesis). Previous research showed the phytochrome effects on plant biomass, resource allocation, and metabolic state under shade [51]. Therefore, the observed low plant biomass under stress is the resulting effect of low photosynthesis and reallocation of metabolites among investigated three tissues. The observed elongation may change the allocation of carbohydrates, which are transferred from leaves to the main stem for elongation. This matches the observed changes in biomass of root, leaf, and stem, which could be a way to balance storing into a biomass and investing in growth under shade stress [52]. Therefore, to achieve high peanut yield in an intercropping system, a variety must have high tolerance to shade and high adaptivity to low light intensity. The observation of very low expression levels of several DEGs, e.g., 0 FPKM, in photosynthesis pathways, and metabolism of sucrose and starch suggests expressional switching off or on, which may play important roles in the shade responsive regulation, but attention should be drawn because those genes’ levels were very low, less than 1 FPKM.

Gene expressional regulation in shade avoidance, especially induced by the low ratio of red and far-red light, has been well studied in model plants. Microarray analysis showed that light signal genes, hormone related genes, and stress induced genes were induced by low ratio of red and far-red light [20]. Another microarray analysis revealed the expressional regulation of genes involved in the metabolism of cell wall carbohydrates, auxin responses, and flavonoids in the stem of tomato [21]. RNA-Seq based transcriptome analyses in other plants like conifers revealed regulations on hormone signaling and pigment biosynthesis under shade [24]. Here, analyses of DEGs also identified enrichments of those reported pathways and more additional pathways, 18 pathways in total in our study, which suggests that shade stress induced systematic changes in pathways and complicated expressional regulation in plants. Of these, under prolonged shade in peanut plants, the two most important pathways, namely photosynthesis and sucrose metabolism, are commonly regulated. An existence of core regulation in light sensitivity and chloroplast metabolism was proposed in an RNA-Seq analysis of dynamic changes of gene expression under shade in *Arabidopsis* [24]. Our finding agrees with that and evidenced a core set of regulated genes in photosynthesis. Besides, we observed core set of expressional regulation in starch and sucrose metabolism under different shade treatments. The enrichment of starch and sucrose metabolism has been reported previously, but has not been investigated in depth [24]. Observed in our study, DEGs regulation indicates that under shade a reduced hydrolysis of sucrose and starch in leaf may be caused by reduced output from the reduced photosynthesis. Together, we conclude that the core set of expressional regulation in photosynthesis and starch and sucrose metabolism could be a common mechanism of shade responses in plants. We also identified DEGs enriched in flavonoid metabolism, which plays role in defense and immunity to diseases. So, it indicates that the shade avoidance may share some common regulation mechanisms with defense and immunity to other stresses. Genes involved in anthocyanin biosynthesis and accumulation were reported to be inhibited under shade [24]. A previous report found that dihydroflavonol 4-reductase involved in anthocyanin biosynthesis pathway was up-regulated in pine, whereas it was downregulated in spruce [24]. Flavanone 3-hydroxylase and leucoanthocyanidin dioxygenase in anthocyanin biosynthesis were down-regulated under shade stress in conifer [24]. Here, in peanut plants experiencing shade stress, we did not observe similar changes in these encoding genes in the flavonoid pathway. We found that two DEGs (gene id *0AN8KE* and *0UU5IV*) encoding isoflavone/4’-methoxyisoflavone 2’-hydroxylase were upregulated while another four DEGs (9WXZ62, IFA20P, QJ0MNA, XS7PLW) encoding the same enzyme were downregulated. Another DEG encoding 2-hydroxyisoflavanone synthase in the flavonoid biosynthesis pathway was upregulated under shade. Therefore, the regulation of flavonoid pathway under shade may be species specific. The detailed role of the expressional regulation of these DEGs under shade is still not clear although it was reported to associated with the anthocyanin biosynthesis [53].

Currently, peanut is used in several intercropping systems with evidence of a positive effect on yield of tall crops [7]. However, the intercropping combination is not good for peanut since we can see a high sensitivity to shade in this investigated peanut cultivar and a reduced yield of peanut seed. This suggests that breeders should pay attention to choosing an appropriate peanut variety with a higher shade tolerance than peanut cultivar Huayu 39 to avoid negative effects of shading on peanut plants.

## 4. Materials and Methods

### 4.1. Plant and Growth Condition

The experiment was conducted at South China Agricultural University (113°15′ E, 23°06′ N) Guangzhou, China during spring in 2019. The peanut cultivar Huayu 39, bred by Shandong Peanut Research Institute, was selected for its lodging resistance and its generally wide use in the actual production in China. One seed was sowed into a pot with a height of 35 cm and a diameter of 40 cm full of 35 kg soil from the 0–20 cm depth of the land surface of Guangzhou (23°09′30″ N, 113°21′52″ E). 

### 4.2. Shade Stress Treatments

Two shadings, 40% and 80% shade, were applied for 15 and 30 days at the seedling stage, flowering stage, and both stages, respectively. The control treatment was done parallelly under natural sunlight. The other peanut plants were put into a shelter covered with different layers of black polyethylene nets, which arrowed 60% and 20% of sunlight to go through, to provide shading termed here as 40% shade and 80% shade. Other field management activities were proceeded according to local agronomic practices.

### 4.3. Length and Biomass Measurements

At the end of each shade treatment, three plants were collected for each treatment. The length and the number of leaves on the main stem were measured. The diameter and length of the third internode counted from the bottom in the main stem were measured. Roots, stems, and leaves were separated and dried at 105 °C for 30 min followed by 80 °C until a constant dry weight was reached. Then dry weight of roots, stems, and leaf leaves were measured.

### 4.4. Analyses of Photosynthesis Parameters

A portable Li-6400 (Li-COR, Lincoln, NE, USA) photosynthesis system, equipped with a red/blue LED light source, was used to measure the net photosynthetic rate, intercellular CO_2_ concentration, stomatal conductance, and transpiration rate of the third leaf (usually called the functional leaf in peanut plant, positioned from the top downwards) between 9:00 and 11:00 a.m. and was operated using a large volume of air with a stable CO_2_ pressure. All measurements were carried out at a photo flux density of 1400 μmol m^−2^ s^−1^ and an ambient CO_2_ concentration of 400 μmol mol^−1^ at 28 °C. The records were made after a stable reading was achieved. The measurement was repeated three times for each plant.

### 4.5. Measurement of Peanut Yield

All peanuts of each plant were harvested at 120 days for yield measurement. We randomly sampled three peanut plants to determine the number of pods per plant. All pods were removed from plants and air-dried until a constant weight was achieved. Then, the 100-pod weight was measured by measuring the weight of a random sample of 100 pods, and the 100-kernel weight in grams was calculated by $\frac{Weight\;of\;kernels}{the\;number\;of\;kernels}\;\times \;100$ for each plant. 

### 4.6. Analysis of Plant Hormone

Peanut leaves were ground with liquid nitrogen and the homogenized material (200 mg) was added into 2 mL of extraction reagent (−4 °C), which consisted of methanol, ultrapure water, and formic acid in the proportion of 15:4:1 (*v:v:v*), according to the previous method [54]. The mixture was vigorously vortexed to obtain a homogenous solution and centrifuged for 5 min with 14000 r min^−1^ at 4 °C. The collected supernatant was dried under vacuum at 35 °C and re-dissolved in 1 ml of complex solution, which consisted of methanol, water, and acetic acid in a proportion of 90:10:0.05 (*v/v/v*), including 10 mmol L^−1^ ammonium acetate. For selection of diagnostic precursor-to-product ion transitions, mixtures of 200 ng/mL of standard compounds dissolved in 50% MeOH with 0.1% HCO_2_H were directly infused into a hybrid triple quadrupole/linear ion reap mass spectrometer (ABI 4000 Q-Traq, Applied Biosystems, Foster City, CA, USA) outfitted with an electrospray ion source using a 1 mL Hamilton syringe pump at a flow rate of 1.2 mL/h. The mixtures of standard compounds were separated by reversed-phase HPLC and analyzed by tandem mass spectrometry in the MRM mode with 20 ms dwell time, 5 ms of pause time between mass range, and 700 bms of settle time for switching polarities. In the “Enhanced Product Ion” scan mode, precursor ions were fragmented with collision energy +25 kV or −25 kV and products in the *m*/*z* range of 50–500 were detected.

### 4.7. RNA Extraction, mRNA Sequencing and Data Deposition

To measure gene regulation in response to shade treatments, RNA-Seq sequencing was used to obtain transcripts and their expression levels. Leaf samples from CK, FS40, FS80, CS40, CS80 treatments were used, and each sample from triplicate experiments was sequenced independently. Briefly, total RNAs were extracted and mRNA was enriched to construct a library for sequencing on an Illumina platform HiSeq X Ten in the paired-end 150 bp followed previous procedures [55]. 8 G bp RNA-Seq reads were generated for each sample. For each treatment or control, three sets of RNA-Seq data were generated, one for each sample. The RNA-Seq reads were deposited and available at the database Short Read Archive at NCBI (https://www.ncbi.nlm.nih.gov) under the master accession number of Bioproject PRJNA629665, and the accession number for each RNA-Seq data is provided in the Supplementary File (Table S4).

### 4.8. Analyses of Transcript Assembly, Abundance, Gene Ontology and Pathway

All RNA-Seq reads were cleaned and mapped into tetraploid peanut *Arachis hypogaea* cv. Tifrunner genome (version 2.0) [34] and transcripts were constructed by using HiSat2 and Stringtie as described previously [39]. The transcript level was calculated as read count and fragment per kilobase per million reads (FPKM). Differentially expressed genes were identified by using DESeq2 with cutoff great than two-fold changes and *p* < 0.05. The DEG-associated gene ontologies (GOs) were enriched by using all GOs of expressed genes as background (hypergeometric, *p* < 0.05). The DEG-associated pathways were analyzed with KAAS against the database KEGG (https://www.genome.jp/kegg/) and then enriched by using the hypergeometric test (*p* < 0.05) [39,40].

### 4.9. Statistical Analysis 

One-way analysis of variance followed by Fisher’s least significant difference test was used to compare different treatment levels with a control for the studied physiological and phenotypic measurements. The analyses were performed with SPSS software (version 24).

## 5. Conclusions

Shade stress reduces the biomass and yield of peanut. The transcriptional regulation under shade stress includes core expressional regulations in peanut plants. Down-regulation of expression of genes in light-harvesting and expressional alteration of genes in photosynthesis reduce photosynthesis activity. Other major regulations are involved in the down-regulation of genes in starch and sucrose metabolism and in the expressional stimulus of growth-promoting genes in plant hormone signal pathways during shade avoidance. Molecular breeding involving selection or manipulation of these genes towards high shade tolerance should guide breeding improvement programs in intercropping practices.

## Figures and Tables

**Table 1:** Effects of shade stress on size of peanut plants.

Treatments			Stem	Stem	Internode	Leaf Number
Stage	Shade	Duration	Length (cm)	Diameter (cm)	Length (cm)	(Count)
Seedling	CK	15	13.8 ± 0.60c	NA	NA	5.67 ± 0.58a
	S40	15	16.4 ± 0.28b	NA	NA	6.67 ± 0.58a
	S80	15	18.0 ± 0.28a	NA	NA	7.00 ± 0.00a
	CK	30	15.9 ± 0.40c	NA	NA	7.33 ± 0.58a
	S40	30	18.7 ± 0.28b *	NA	NA	7.67 ± 0.58a
	S80	30	20.2 ± 0.14a *	NA	NA	8.00 ± 0.00a *
Flowering	CK	15	15.8 ± 0.23c	0.45 ± 0.00a	2.13 ± 0.25c	9.00 ± 0.00a
	S40	15	21.2 ± 0.75b	0.40 ± 0.01b	2.83 ± 0.21b	11.33 ± 0.57a
	S80	15	28.3 ± 0.35a	0.33 ± 0.01c	3.70 ± 0.10a	12.50 ± 2.12a
	CK	30	17.9 ± 0.20c	0.34 ± 0.01a	2.38 ± 0.17c	10.33 ± 0.57a
	S40	30	24.6 ± 0.47b*	0.32 ± 0.01b *	3.41 ± 0.17b	11.85 ± 0.30a
	S80	30	30.8 ± 0.66a	0.29 ± 0.01c	4.07 ± 0.16a	13.10 ± 1.21a
Seedling	CK	60	17.9 ± 0.20c	0.34 ± 0.06a	2.38 ± 0.17c	10.33 ± 0.57b
and	S40	60	31.4 ± 0.38b	0.30 ± 0.01b	3.47 ± 0.22b	15.00 ± 1.00a
flowering	S80	60	39.2 ± 0.54a	0.28 ± 0.01c	4.33 ± 0.22a	14.50 ± 0.71a

Note: Data represent the mean ± standard error from three experimental replicates, each with three plants. Letters a, b, c after the value represent a statistically significant difference (*p* < 0.05) within a stage as determined by the Least Significant Difference test. * represents a significant difference (*p* < 0.05) between different durations of 15 days and 30 days at the same stage. S40 and S80 represent 40% and 80% shade, which allows 60% and 20% natural sunlight to go through. The internode refers to the longest internode at the third position counting from the bottom. NA represents that the data is not available.

**Table 2:** Effects of shade stress on peanut yield components.

Stage	Shade	Duration (Day)	Pods Per Plant	100-Pod Weight (g)	100-Kernel Weight (g)
Seedling	CK	15	15.30 ± 0.90a	114.80 ± 0.71a	64.85 ± 1.07a
	S40	15	10.00 ± 0.60b	106.69 ± 1.41b	55.81 ± 0.60b
	S80	15	8.70 ± 0.90b	101.25 ± 2.15c	44.93 ± 1.14c
	CK	30	15.30 ± 0.90a	114.80 ± 0.70a	64.85 ± 1.07a
	S40	30	5.70 ± 0.70b **	100.35 ± 0.54b *	44.87 ± 1.09b **
	S80	30	5.30 ± 0.90b *	96.58 ± 0.74c	41.06 ± 1.04c
Flowering	CK	15	15.30 ± 0.90a	114.80 ± 0.70a	64.85 ± 1.07a
	S40	15	6.00 ± 1.00b	95.00 ± 2.22b	49.58 ± 1.19b
	S80	15	5.3 ± 0.30b	88.87 ± 1.74c	39.67 ± 1.45c
	CK	30	15.30 ± 0.90a	114.80 ± 0.71a	64.85 ± 1.07a
	S40	30	2.30 ± 0.90b *	91.05 ± 1.17b	40.30 ± 1.44b *
	S80	30	2.70 ± 0.70b *	84.46 ± 1.17c	36.14 ± 0.57c
Seedling	CK	60	15.30 ± 0.90a	114.80 ± 0.71a	64.85 ± 1.07a
and	S40	60	1.30 ± 0.90b	81.24 ± 1.53b	32.14 ± 1.68b
Flowering	S80	60	1.30 ± 0.70b	67.67 ± 1.46c	28.66 ± 0.62b

Note: Data represent the mean ± standard error from three experimental replicates, each with three plants. Letters a, b, and c after the value represent the statistically significant difference (*p* < 0.05) between any two conditions within a stage as determined by the Least Significant Difference test. * represents a significant difference (*p* < 0.05) and ** represents a significant difference (*p* < 0.01) between different treating durations of 15 days and 30 days. S40 and S80 represent 40% and 80% shade, which allows 60% and 20% natural sunlight to go through.

**Table 3:** The shade stress-induced DEGs encoding light-harvesting chlorophyll complexes.

	Gene_ID	Symbol ^$^	Description of Encoded Proteins	K_ID ^#^
1	14YEDZ	CAB13	light-harvesting complex II chlorophyll a/b binding protein 3	K08914
2	AFZN0R	CAB13	light-harvesting complex II chlorophyll a/b binding protein 3	K08914
3	BU7NMG	CAB13	light-harvesting complex II chlorophyll a/b binding protein 3	K08914
4	JKC32H	CAB13	light-harvesting complex II chlorophyll a/b binding protein 3	K08914
5	QXM1B7	CAB3	light-harvesting complex II chlorophyll a/b binding protein 3	K08912
6	YZ06AV	CAB3	light-harvesting complex II chlorophyll a/b binding protein 3	K08912
7	BTM1YE	CAB6A	light-harvesting complex II chlorophyll a/b binding protein 3	K08907
8	NKTC04	CAB6A	light-harvesting complex II chlorophyll a/b binding protein 3	K08907
9	9BXG3M	CAB7	light-harvesting complex I chlorophyll a/b binding protein 2	K08908
10	AAY07D	CAB7	light-harvesting complex I chlorophyll a/b binding protein 2	K08908
11	EB48YM	CAB7	light-harvesting complex I chlorophyll a/b binding protein 2	K08908
12	66SDN0	CAB8	light-harvesting complex I chlorophyll a/b binding protein 3	K08909
13	86F2TK	CAB8	light-harvesting complex I chlorophyll a/b binding protein 3	K08909
14	4KW7H9	CAP10A	light-harvesting complex II chlorophyll a/b binding protein 6	K08917
15	JVEI5Y	CAP10A	light-harvesting complex II chlorophyll a/b binding protein 6	K08917
16	N8DZQ8	LHCA-P4	light-harvesting complex I chlorophyll a/b binding protein 4	K08910
17	RLTX4G	LHCA-P4	light-harvesting complex I chlorophyll a/b binding protein 4	K08910
18	YZB02J	LHCA6	light-harvesting complex I chlorophyll a/b binding protein 2	K08908
19	6DQ39T	LHCB4.1	light-harvesting complex II chlorophyll a/b binding protein 4	K08915
20	CVJ130	LHCB4.1	light-harvesting complex II chlorophyll a/b binding protein 4	K08915
21	2C7VNA	LHCB4.3	light-harvesting complex II chlorophyll a/b binding protein 4	K08915
22	63GP52	LHCB4.3	light-harvesting complex II chlorophyll a/b binding protein 4	K08915
23	8LQR2U	LHCB5	light-harvesting complex II chlorophyll a/b binding protein 5	K08916
24	J9AJ01	LHCB5	light-harvesting complex II chlorophyll a/b binding protein 5	K08916

Note: the symbol $ of the gene was extracted from KEGG (www.genome.jp/kegg/) based on the best match to the homologous reference genes in the database KEGG. # K_ID represents the id number of homologous at the pathway database KEGG.

**Table 4:** The shade stress-induced DEGs and annotations in photosynthesis pathway.

Gene_ID	Symbol ^$^	Description of Encoded Proteins	K_ID ^#^
AF7BT4	atpA	ATP synthase F1, alpha subunit	K02111
487MWI	atpB	ATP synthase, F1 beta subunit	K02112
KPH33C	atpI	ATP synthase subunit A	K02108
26BATJ	Os03g0784700	Ferredoxin-NADP reductase family protein	K02641
Z0BJS7	petA	chloroplast envelope membrane protein-like isoform X2 [Glycine max]	K02634
B5AM0N	petB	photosynthetic electron transfer B	K02704
GZ00NU	petD	photosynthetic electron transfer D	K02637
E2679H	PETH	ferredoxin-NADP(+)-oxidoreductase 1	K02641
ZQ3F6U	PETH	ferredoxin-NADP(+)-oxidoreductase 1	K02641
WM01M4	PNSL2	oxygen-evolving enhancer protein	K08901
3I3T69	psaA	photosystem I P700 chlorophyll A apoprotein	K02689
7VZ02V	psaA	photosystem I P700 chlorophyll A apoprotein	K02689
R5AFBV	psaA	photosystem I P700 chlorophyll A apoprotein	K02690
UAEM0K	psaB	photosystem I P700 chlorophyll A apoprotein	K02690
QW0L8Q	PSAE-1	photosystem I reaction center subunit IV A	K02693
VHAI7W	PSAF	photosystem I reaction center subunit III	K02694
4PD0PP	PSAG	photosystem I reaction center subunit V	K08905
71HCYD	PSAH2	photosystem I reaction center subunit VI	K02695
YBSA05	PSAH2	photosystem I reaction center subunit VI	K02695
9042LP	PSAK	photosystem I reaction center subunit X psaK	K02698
ZS1M1K	PSAK	photosystem I reaction center subunit X psaK	K02698
18YQBF	PSAL	photosystem I reaction center subunit XI	K02699
0R479N	psbA	photosystem II protein D1 [Glycine max]	K02703
0W708Z	psbB	photosystem II CP47 chlorophyll A apoprotein	K02704
8A6KBK	psbC	Photosystem II chlorophyll-binding protein CP43	K02705
92HTUM	psbC	photosystem II CP43 chlorophyll apoprotein	K02705
49ZYT6	PSBO	photosystem II oxygen-evolving enhancer protein	K02716
30A6BG	PSBP	23kDa polypeptide of the oxygen evolving complex of photosystem II	K02717
L9FPHH	PSBS	photosystem II 22 kDa protein, chloroplastic-like [Glycine max]	K03542
QP7RRL	PSBS	photosystem II 22 kDa protein, chloroplastic-like [Glycine max]	K03542
J2LHWB	PSBY	photosystem II core complex family psbY protein	K02723
CW3FX9	SEND33	ferredoxin 1	K02639
I8TN6N	YMF19	ATPase subunit 8 (mitochondrion) [Glycine max]	K02109

Note: $ the symbol of the gene was extracted from KEGG (www.genome.jp/kegg/) based on the best match to the homologous reference genes in the database KEGG. # K_ID represents the id number of homologous at the pathway database KEGG (www.genome.jp/kegg/).

**Table 5:** Shade commonly regulated DEGs in starch and sucrose metabolism.

Gene ID	Encoded Enzyme	KEGG_ID	Abbreviation	Enzyme	Function
L6ZHES	glucose-1-phosphate adenylyltransferase 1	K05349	AGPS1	[EC:3.2.1.21]	Glycosidases that hydrolyse O- and S-glycosyl compounds
V374FJ	glucose-1-phosphate adenylyltransferase 1	K05349	AGPS1	[EC:3.2.1.21]	Glycosidases that hydrolyse O- and S-glycosyl compounds
PX7TTT	glucose-1-phosphate adenylyltransferase	K00975	glgC	[EC:2.7.7.27]	Transferring phosphorus-containing groups for glycogen synthesis
4Y2508	beta-amylase 1	K01177	BAM1	[EC:3.2.1.2]	hydrolyse O- and S-glycosyl compounds
4Z6VDH	beta glucosidase 15	K01188	BGLU12	[EC:2.4.1.1]	hydrolyse O- and S-glycosyl compounds
M8S3TB	beta glucosidase 15	K01188	BGLU12	[EC:2.4.1.1]	hydrolyse O- and S-glycosyl compounds
YDQ82R	beta glucosidase 15	K01188	BGLU12	[EC:2.4.1.1]	hydrolyse O- and S-glycosyl compounds
IXM19M	glycogen phosphorylase 1-like isoform X1	K00688	glpV	[EC:2.4.1.1]	Glycogen degradation, glycogen => glucose-6P
0Q5BJB	beta-fructofuranosidase 5; or Invertase	K01193	INV	[EC:3.2.1.26]	hydrolyse O- and S-glycosyl compounds, e.g. sucrose
4VZ14Z	sucrose synthase 4	K00695	SS	[EC:2.4.1.13]	sucrose synthesis and hydrolysis
0FY2NM	trehalose-6-phosphate phosphatase	K01087	TPS	[EC:3.1.3.12]	catalyze trehalose-6p to trehalose
G2WE73	trehalose-6-phosphate phosphatase	K01087	TPS	[EC:3.1.3.12]	catalyze trehalose-6p to trehalose

Note: # KEGG represents the pathway database KEGG (www.genome.jp/kegg/).

**Table 6:** Effects of shade stress on biomass of peanut plants.

Treatments			Root		Stem		Leaf		Pod	
Stage	Shade	Duration	Dry Weight (g)	Ratio (%)	Dry Weight (g)	Ratio (%)	Dry Weight (g)	Ratio (%)	Dry weight (g)	Ratio (%)
Seedling	CK	15	0.19 ± 0.03a	8.39	0.55 ± 0.07a	24.10	1.55 ± 0.48a	67.51	NA	NA
	S40	15	0.16 ± 0.05a	12.89	0.42 ± 0.00a	34.46	0.64 ± 0.08b	52.64	NA	NA
	S80	15	0.07 ± 0.01b	13.64	0.15 ± 0.01b	30.87	0.27 ± 0.01b	55.49	NA	NA
	CK	30	0.63 ± 0.07a	6.6.1	3.74 ± 0.29a	39.30	5.15 ± 0.43a	54.08	NA	NA
	S40	30	0.31 ± 0.02b *	5.06	2.67 ± 0.10b **	43.67	3.14 ± 0.45b **	51.26	NA	NA
	S80	30	0.28 ± 0.03b **	9.53	1.11 ± 0.15c *	37.26	1.58 ± 0.36c *	53.21	NA	NA
Flowering	CK	15	0.63 ± 0.07a	6.62	3.74 ± 0.29a	39.29	5.15 ± 0.43a	54.10	NA	NA
	S40	15	0.44 ± 0.02b	5.25	3.52 ± 0.17a	42.00	4.42 ± 0.16a	52.74	NA	NA
	S80	15	0.26 ± 0.00c	4.68	2.21 ± 0.13b	39.82	3.08 ± 0.12b	55.50	NA	NA
	CK	30	0.68 ± 0.05a	6.07	3.78 ± 0.28a	33.72	5.31 ± 0.56a	47.37	1.44 ± 0.12a	12.85
	S40	30	0.46 ± 0.05b	7.11	2.69 ± 0.24b *	41.58	2.88 ± 0.34b *	44.51	0.44 ± 0.07b	6.80
	S80	30	0.31 ± 0.06c	6.95	1.71 ± 0.23c	38.34	2.19 ± 0.19c *	49.10	0.35 ± 0.10b	5.61
Seedling	CK	60	0.68 ± 0.05a	6.07	3.78 ± 0.28a	33.72	5.31 ± 0.56a	47.37	1.44 ± 0.12a	12.85
and	S40	60	0.33 ± 0.13b	6.52	2.36 ± 0.17b	46.64	2.10 ± 0.09b	41.50	1.07 ± 0.07b	5.34
flowering	S80	60	0.16 ± 0.02c	8.12	0.86 ± 0.15c	43.65	0.95 ± 0.04c	48.22	0.00 ± 0.00c	0.00

Note: Data represent the mean ± standard error from three experimental replicates, each with three plants. Letters a, b, c after the value represent the statistically significant difference (*p* < 0.05) between any two conditions within a stage as determined by the Least Significant Difference test. * represents a significant difference (*p* < 0.05) and ** represents a significant difference (*p* < 0.01) between different treating durations of 15 days and 30 days. The ratio is the percent of the specific biomass in total tested biomass. S40 and S80 represent 40% and 80% shade, which allows 60% and 20% natural light to go through, respectively. CK represents the control under natural light at a corresponding developmental stage. NA represents the data is not available.

## Supplementary Materials

Supplementary materials can be found at https://www.mdpi.com/1422-0067/21/15/5284/s1; Table S1: Affected pathways involved by common DEGs induced by all shade treatments; Table S2: The FPKM of differentially expressed genes in photosynthesis pathway; Table S3: The FPKM of differentially expressed genes in starch and sucrose metabolism; Table S4: RNA-Seq data accession, sample name and treatment at NCBI; Data S1: The DEGs information and expression level between control and CS40 (CK-vs-CS40.DEG_FPKM.xls); Data S2: The DEGs information and expression level between control and CS80 (File: CK-vs-CS80.DEG_FPKM.xls); Data S3: The DEGs information and expression level between control and FS40 (File:CK-vs-FS40.DEG_FPKM.xls); Data S4: The DEGs information and expression level between control and FS80 (File:CK-vs-FS80.DEG_FPKM.xls); Data S5: Common DEGs across all shade conditions
