# Supplementary material for: Shade Effects on Peanut Yield Associate with Physiological and Expressional Regulation on Photosynthesis and Sucrose Metabolism
Source: Int J Mol Sci. 2020 Jul 25;21(15):5284. doi: 10.3390/ijms21155284 (PMC7432592; doi:10.3390/ijms21155284)
Supplement: Supplementary file 1 [file ijms-21-05284-s001.zip › Supplementary information/Supplementary information20200701.docx]

**Supplementary information**

Shade effects on peanut yield associate with physiological and expressional regulation on photosynthesis and sucrose metabolism

Tingting Chen^1†^, Huajian Zhang^1†^, Ruier Zeng^1^, Xinyue Wang^1^, Luping Huang^1^, Leidi Wang^1^, Xuewen Wang^2, *^ and Lei Zhang^1, *^

^1^ Guangdong Key Laboratory of Plant Molecular Breeding, State Key Laboratory for Conservation and Utilization of Subtropical Agro-Bioresources, College of Agriculture, South China Agricultural University, Guangzhou, 510642, China; chentingting@scau.edu.cn, 15099976174@163.com, ruierzeng@126.com, wangxinyuescau@163.com, lupinghuang2019@163.com, wangld@mail.iap.ac.cn

^2^ Department of Genetics, University of Georgia, Athens, GA, 30602, USA; xwwang@uga.edu

***** Correspondence: xwwang@uga.edu (X.W.); zhanglei@scau.edu.cn (L.Z.)

^†^ These authors contributed equally to this work.

Received: date; Accepted: date; Published: date

**Supplementary Table**

Table S 1 Affected pathways involved by common DEGs induced by all shade treatments

Table S 2 The FPKM of differentially expressed genes in photosynthesis pathway

Table S 3 The FPKM of differentially expressed genes in starch and sucrose metabolism

Table S 4 RNA-Seq data accession, sample name and treatment at NCBI

**Supplementary data**

**Data S1 The DEGs information and expression level between control and CS40**

**file:** CK-vs-CS40.DEG_FPKM.xls

**Data S2 The DEGs information and expression level between control and CS80**

CK-vs-CS80.DEG_FPKM.xls

**Data S3 The DEGs information and expression level between control and FS40**

CK-vs-FS40.DEG_FPKM.xls

**Data S4 The DEGs information and expression level between control and FS40**

CK-vs-FS80.DEG_FPKM.xls

**Data S5 Common DEGs across all shade conditions** of CS40, CS80, FS40, and FS80 relative to corresponding natural light. Each ID is separated by a semi-common. DEG ID list:

0F111G;5H5XYM;HG1KKP;E5BVVA;ALW2B1;E35BH4;FM2N1A;RR76TU;J41PYN;9E4VJ9;T25X4W;P3CRQW;NG49FS;2NH314;QWH41Z;79B99S;MVB9VE;QKV9FM;SLB9HH;HYLU9Z;AZ9RHT;XJ8JS0;B8KEMK;G2WE73;A5ASCL;B55RN5;T4QRY3;Q6RBV6;GMWG2V;MSTRG.8042;0C8NZA;FXNF4I;MSTRG.37236;4Y3SQC;X05KEZ;KBQI3L;E8N93X;XZH3XT;FDV79C;YH7XEC;XJ0R0A;B1GHBY;CPL9KJ;4VZ14Z;D236HJ;7J7FTE;NH86F6;84G6ZA;80ML42;H3EIYM;B35K79;RXWH3W;3J2MYH;C9KM7P;N8DZQ8;I192KR;H4HZRI;NB9ZER;1YR532;JF12SQ;A37Y50;6X9KLT;0T4V2C;M0AD1K;ZQ7JRA;CM90T6;I2HAHG;E2AJGQ;YNQ4ZP;J2GFEF;Y5BWXR;H39N2I;E862VI;7E5NA9;L3KDU2;31BJ59;4UTS2Z;T45XFJ;L6NNIX;45B7J4;ZDDP39;UM7XUK;N1LTPI;HW5QP5;M4EJC3;21N9MM;WT21TW;CMKD6L;95S5ZX;2LG1YU;MSTRG.9493;5BA4ES;5CZ8DH;240DSL;VDQ3GL;C70Y3X;FH6P3F;JBC01Z;MSTRG.3509;T13AP3;LUT726;X9M6WH;MSTRG.39753;M8S3TB;RLTX4G;7N8JZK;7VP0DB;VI6I29;QF8GL7;JS39C6;4LP3QH;A6Q8V1;SB835A;LR2QH7;MSTRG.34307;VI1C6C;3IB3IU;4Y2508;6HIR6Y;D01KFK;VZ3VQN;0V6YW0;AGD9YF;2PL4X0;4Z6VDH;9C0JE8;G8PY79;EY5UDZ;12G0NV;T01IT8;YLB5I5;MSTRG.3731;IK43K5;3U22LJ;UG8EST;1W4SSV;TJMK61;RQ5XX8;185HYR;WYU9FD;A6N0MA;BCI2ZL;VIU8IP;V31KAL;M2PBL6;RSU8B2;47SX7B;5PE8VQ;IXM19M;6Q641Y;SZ7SGM;R2NPVK;DC259S;H9ZQVQ;L0R21L;WLS118;SWNJ62;KRA1N7;BU0FM1;5PJB70;LXJ7K2;D9Q2X2;ZE0PKC;GDD7VZ;KNQB38;XPK4NL;Y2U02I;NKRN6N;JA5933;89K6QE;BQEK3T;CDL3P0;JNL70L;3YF6JX;I649W8;E945XK;VAJ8HR;V97YAG;UC02TM;HZDF69;N9MUEN;M16IE8;74CD3Z;MSK3BR;GZ7CAY;B68HGA;E2GMYT;C0LV86;ZBZ102;P8DJ8N;8X5LLL;AS0BXY;MSTRG.341;FMU3NZ;5QD1DC;KTW86Y;Z2HVA4;98EFWE;RL0B30;YJIY2K;F2RES5;Q9WW27;P4M8HI;E3Q78B;HDLN9F;YS4IQ1;X5MG7Q;780T1G;IJP2R9;52XQ2N;PNDG01;MSTRG.31950;GVHP98;27E6QC;UU73T8;MSTRG.34280;M165BX;88MUGQ;0R0FR4;PN5ECJ;U8I7FL;G0DDCQ;CIN1QH;98HCYL;FG3TV9;RMT1KV;CP2NGN;W4CKNF;QRF4HN;AQHB6D;X02P63;77E9KA;MSTRG.12666;EMW6AM;ALL5TI;37XEK7;LH9WUE;K8H9R8;I5R24F;8JHM47;R1FZ8C;ISPD6P;ATC700;58NQ7F;ZU9B18;A9DQ77;7NC073;39MK8P;UXKX2B;09991I;Z95P5D;PN33QT;A4DTXZ;R8DMLF;0Q5BJB;TNW0AK;X6QVVL;0NW365;P2PL73;F8Q1RV;JGYC2J;BLVJ4U;RBAX1J;HNM84L;U9RBLI;HY7WV5;G7VI5W;B9PVT7;H5ITG5;RQU8DI;Q4PIE3;8VMK61;KW18SN;MSTRG.37140;QLUK9J;MSTRG.32109;NCR4KV;MSTRG.40952;HU2V9F;VAE62H;75Z370;849EC4;B0G4GL;SCN40X;SDSH2P;D43PH9;XK2NFV;3BDP96;ALJH5D;R0KKYG;B1R1KQ;AJ9HGA;XH5ZLH;Q1XFSF;IE3GQ3;H4RKER;C0B70Q;Q5MPVN;TTC3KU;PA3NAF;MSTRG.17004;MSTRG.15474;2C1RPT;XXTB4R;A9G6ZW;67NNK3;HQ8G2J;1RK1ZX;BBW5IA;6374NN;4TV0N2;NDX0CI;Z1FW9R;W54VUE;3H9TDX;KQ3B15;7A9T8X;D4N0FV;3N6FKX;8G8R7R;I1HNZH;L13VJ0;I4AHF2;V739AW;4Y1607;M9SN56;AB2F1A;H003I6;54FW37;03QWZ4;CWZU13;NAPQ43;HP76IN;84IRST;LRF6TA;5P912R;642FWA;BD5KCN;VJD5NB;9KBM2K;069QKB;N2JMJ4;J4T639;U3X98C;T9R3PD;GADS7N;P7J5GP;F87Z6X;AZ3QQS;MSTRG.36858;YDQ82R;HLS3VZ;QAC8GG;21R0E2;V0UCAK;AU0S52;MU7GXS;U5HRAC;HKX9QT;LX021I;4LI2NJ;00VVAL;MSTRG.26682;ILN3K9;42CZAS;57U25Z;0NK0N0;SY7QJV;G0ML1P;57AKVM;S5DSK4;FX71XI;A0U9FM;4U2MDP;MSTRG.9318;VKVT6A;F3E022;SBZM2C;KHE3JK;MSTRG.34449;EBVI20;2ZBI42;K44PA8;4WXU8P;38WMVK;NBP004;P45MK1;Z92S4S;YX4IN3;BHNX0U;JVJR89;HE8J5U;4749WV;24ZZ8V;GA8N54;V374FJ;S82084;0BPR43;2R4GL5;WE5WNX;08FCR9;M6NE4G;TC7Y0P;2J0KXT;S52WLR;AUY4CV;3F0XG2;VI8BIT;1CEJ34;PEQF49;HE9KP0;NWD6TW;1W5D66;MSTRG.17268;M5E8P8;PF3CMY;72HQJ5;PF6WY1;BMYY0J;BX8PA5;U3WWWH;SG819P;TJC463;71GWZM;SI8X9Q;WX7AU9;MSTRG.21401;PX7TTT;QU2PQ7;6P73LQ;8GI8KI;K25JBG;L0RB90;EQ2CTR;711CF1;L6USB4;17Q9T0;HAI9AH;EJ77H8;BAG535;L50WS8;XR90DA;F1ZF7M;LV620I;I5UYY8;PZQY06;NALL13;GVDE30;MSTRG.9277;GJFA3Z;VW08M3;90Q9AE;T2K6QT;DZTW61;0UT8YS;6C4W0P;5913QL;MSTRG.9280;MSTRG.18750;5M2QCV;Q95PA7;U8F7KB;ED5BBM;4NL1XF;MX4DT2;27RHGS;FTX6XU;H8QW84;0FY2NM;PV9TPZ;MA69BJ;M7A5MM;DBME17;L6ZHES;IWH7X5;RSK4RP;S6H019;77K1W8;C516TH;MSTRG.38677;V960AN;8I98WG;DW7R17;R6L1G9;79XMYU;1KM4NQ;XVCK9B;I8F8BQ;3U55TZ;KMJ8U9;6P3KM7;KDG5NW;GT8KW4;5RL6NX;5IT73U;Z3NZLL;MSTRG.34279;JZ1PRS;MSTRG.46146;AWX4M7;HAV8AB;Q6HX7B;6J3MKV;9WXZ62;MQY6TT;FK15VW;AXAR8E;TUBV7Y;2T9GCU;WH2MYH;ZZBS45;18QESW;85MSRM;7YIL9E;JA012F;EZ0RLS;22VPXE;ATM50A;U2UX9Q;GL9F8W;TR389G;HWY8JN;K5MJK0;VY5ZYI;Y6ZEHB;68JQQM;R8ZZ7K;MSTRG.10909;WS4P7I;U93XWB;D0T0UQ;W0TF53;VT3Q0W;S2WTSA;7G8F9G;MZFE1X;F24DAH;PP2Z6E;MSTRG.13576;1LGD4F;MNN997;V7KKLB;D18R6M;PNL0JR;B8JBQ4;MX4LCS;PPBW4B;8F7PE4;ICVP6R;7K3ENV;QNIY5E;QQT54I;YFEH4R;ZM6ZIL;FTVA6C;I3R247;0C34XQ;EY0R4L;2P95X7;AMV68J

**Supplemental table**

Table S 1 Affected pathways involved by common DEGs induced by all shade treatments

| KEGG ID | Pathway | Involved by DEGs | Involved by genes | P value^#^ |  |
| --- | --- | --- | --- | --- | --- |
| ko01100 | Metabolic pathways | 73 | 6506 | 2.1E-03 | * |
| ko01110 | Biosynthesis of secondary metabolites | 55 | 3508 | 4.3E-06 | * |
| ko00940 | Phenylpropanoid biosynthesis | 16 | 458 | 1.7E-06 | * |
| ko04075 | Plant hormone signal transduction | 15 | 796 | 2.0E-03 | * |
| ko00500 | Starch and sucrose metabolism | 12 | 457 | 4.0E-04 | * |
| ko04016 | MAPK signaling pathway - plant | 12 | 438 | 2.8E-04 | * |
| ko00941 | Flavonoid biosynthesis | 11 | 158 | 1.0E-07 | * |
| ko04712 | Circadian rhythm - plant | 9 | 182 | 2.2E-05 | * |
| ko00561 | Glycerolipid metabolism | 7 | 259 | 4.9E-03 | * |
| ko00460 | Cyanoamino acid metabolism | 4 | 173 | 4.2E-02 | * |
| ko00920 | Sulfur metabolism | 4 | 89 | 5.8E-03 | * |
| ko00591 | Linoleic acid metabolism | 4 | 56 | 1.2E-03 | * |
| ko00750 | Vitamin B6 metabolism | 4 | 29 | 9.3E-05 | * |
| ko00760 | Nicotinate and nicotinamide metabolism | 3 | 72 | 0.02 | * |
| ko00196 | Photosynthesis - antenna proteins | 2 | 33 | 0.03 | * |
| ko00902 | Monoterpenoid biosynthesis | 2 | 33 | 0.03 | * |
| ko00944 | Flavone and flavonol biosynthesis | 2 | 26 | 0.02 | * |
| ko03010 | Ribosome | 1 | 862 | 0.01 | * |
| ko00740 | Riboflavin metabolism | 2 | 56 | 0.07 |  |
| ko00380 | Tryptophan metabolism | 3 | 152 | 0.10 |  |
| ko01040 | Biosynthesis of unsaturated fatty acids | 2 | 80 | 0.11 |  |
| ko01200 | Carbon metabolism | 5 | 879 | 0.12 |  |
| ko00480 | Glutathione metabolism | 4 | 275 | 0.12 |  |
| ko00860 | Porphyrin and chlorophyll metabolism | 3 | 173 | 0.12 |  |
| ko00330 | Arginine and proline metabolism | 3 | 178 | 0.12 |  |
| ko04626 | Plant-pathogen interaction | 8 | 899 | 0.14 |  |
| ko00564 | Glycerophospholipid metabolism | 4 | 309 | 0.14 |  |
| ko00052 | Galactose metabolism | 3 | 194 | 0.14 |  |
| ko00073 | Cutin, suberine and wax biosynthesis | 2 | 94 | 0.14 |  |
| ko01230 | Biosynthesis of amino acids | 6 | 681 | 0.16 |  |
| ko03018 | RNA degradation | 1 | 337 | 0.17 |  |
| ko00190 | Oxidative phosphorylation | 3 | 541 | 0.17 |  |
| ko04144 | Endocytosis | 5 | 520 | 0.17 |  |
| ko00520 | Amino sugar and nucleotide sugar metabolism | 4 | 359 | 0.17 |  |
| ko00965 | Betalain biosynthesis | 1 | 25 | 0.17 |  |
| ko00909 | Sesquiterpenoid and triterpenoid biosynthesis | 2 | 113 | 0.17 |  |
| ko03015 | mRNA surveillance pathway | 1 | 313 | 0.19 |  |
| ko04141 | Protein processing in endoplasmic reticulum | 4 | 529 | 0.19 |  |
| ko03040 | Spliceosome | 4 | 449 | 0.20 |  |
| ko00270 | Cysteine and methionine metabolism | 3 | 273 | 0.20 |  |
| ko00592 | alpha-Linolenic acid metabolism | 2 | 134 | 0.21 |  |
| ko00901 | Indole alkaloid biosynthesis | 1 | 34 | 0.22 |  |
| ko04146 | Peroxisome | 3 | 309 | 0.22 |  |
| ko00010 | Glycolysis / Gluconeogenesis | 3 | 410 | 0.22 |  |
| ko00710 | Carbon fixation in photosynthetic organisms | 1 | 272 | 0.23 |  |
| ko00040 | Pentose and glucuronate interconversions | 2 | 322 | 0.25 |  |
| ko04070 | Phosphatidylinositol signaling system | 2 | 185 | 0.26 |  |
| ko04145 | Phagosome | 2 | 296 | 0.26 |  |
| ko00562 | Inositol phosphate metabolism | 2 | 197 | 0.26 |  |
| ko00260 | Glycine, serine and threonine metabolism | 2 | 210 | 0.27 |  |
| ko01212 | Fatty acid metabolism | 2 | 217 | 0.27 |  |
| ko00630 | Glyoxylate and dicarboxylate metabolism | 1 | 237 | 0.27 |  |
| ko00945 | Stilbenoid, diarylheptanoid and gingerol biosynthesis | 1 | 59 | 0.30 |  |
| ko00943 | Isoflavonoid biosynthesis | 1 | 60 | 0.31 |  |
| ko00531 | Glycosaminoglycan degradation | 1 | 61 | 0.31 |  |
| ko00130 | Ubiquinone and other terpenoid-quinone biosynthesis | 1 | 198 | 0.32 |  |
| ko00906 | Carotenoid biosynthesis | 1 | 72 | 0.33 |  |
| ko00950 | Isoquinoline alkaloid biosynthesis | 1 | 74 | 0.34 |  |
| ko00900 | Terpenoid backbone biosynthesis | 1 | 177 | 0.34 |  |
| ko00350 | Tyrosine metabolism | 1 | 172 | 0.34 |  |
| ko00904 | Diterpenoid biosynthesis | 1 | 81 | 0.35 |  |
| ko00360 | Phenylalanine metabolism | 1 | 158 | 0.35 |  |
| ko00100 | Steroid biosynthesis | 1 | 142 | 0.36 |  |
| ko00310 | Lysine degradation | 1 | 130 | 0.37 |  |
|  | Back_ground total | 762 | 91204 |  |  |

# note: the significance was examined with hypergeometric test. * represent P < 0.05.

Table S 2 The FPKM of differentially expressed genes in photosynthesis pathway

| Pathway ID | Gene_id | CK_CS40 | CS40 | CK_CS80 | CS80 | CK_FS40 | FS40 | CK_FS80 | FS80 |
| --- | --- | --- | --- | --- | --- | --- | --- | --- | --- |
| 195 | AF7BT4 | 7.8 | 5.5 | 7.8 | 17.7 | 7.8 | 4 | 7.8 | 11.1 |
| 195 | 487MWI | 6.3 | 7.6 | 6.3 | 16 | 6.3 | 4.2 | 6.3 | 8.6 |
| 195 | KPH33C | 2.5 | 2.6 | 2.5 | 10.4 | 2.5 | 1.8 | 2.5 | 5 |
| 195 | 26BATJ | 14.1 | 15.2 | 14.1 | 19.4 | 14.1 | 19.9 | 14.1 | 29.9 |
| 195 | Z0BJS7 | 2.6 | 2.7 | 2.6 | 8.9 | 2.6 | 1.8 | 2.6 | 4.6 |
| 195 | B5AM0N | 14.5 | 12.6 | 14.5 | 31.6 | 14.5 | 8.7 | 14.5 | 24.4 |
| 195 | GZ00NU | 8.2 | 8.7 | 8.2 | 31.9 | 8.2 | 5.5 | 8.2 | 21.1 |
| 195 | E2679H | 6.4 | 6.1 | 6.4 | 2.4 | 6.4 | 7.7 | 6.4 | 4.9 |
| 195 | ZQ3F6U | 184.5 | 210.2 | 184.5 | 108.7 | 184.5 | 175.8 | 184.5 | 89.6 |
| 195 | WM01M4 | 1.8 | 2.9 | 1.8 | 7.9 | 1.8 | 1.4 | 1.8 | 1.6 |
| 195 | 3I3T69 | 37.4 | 36.7 | 37.4 | 85.5 | 37.4 | 23.4 | 37.4 | 54.5 |
| 195 | 7VZ02V | 0 | 0 | 0 | 0.3 | 0 | 0 | 0 | 0 |
| 195 | R5AFBV | 1.4 | 1.2 | 1.4 | 3.9 | 1.4 | 0.8 | 1.4 | 2.4 |
| 195 | UAEM0K | 33.5 | 31.4 | 33.5 | 78.7 | 33.5 | 20.7 | 33.5 | 51.5 |
| 195 | QW0L8Q | 134.3 | 92.1 | 134.3 | 85.5 | 134.3 | 82.6 | 134.3 | 51.9 |
| 195 | VHAI7W | 363.2 | 377.4 | 363.2 | 212.9 | 363.2 | 319.5 | 363.2 | 167.5 |
| 195 | 4PD0PP | 1259.3 | 1571.7 | 1259.3 | 776.2 | 1259.3 | 1226.4 | 1259.3 | 603.6 |
| 195 | 71HCYD | 316.6 | 305.7 | 316.6 | 185.8 | 316.6 | 244.4 | 316.6 | 126.1 |
| 195 | YBSA05 | 635.1 | 533 | 635.1 | 268.9 | 635.1 | 461.4 | 635.1 | 219.3 |
| 195 | 9042LP | 201 | 157.2 | 201 | 144.6 | 201 | 161.1 | 201 | 77.5 |
| 195 | ZS1M1K | 132.4 | 104.4 | 132.4 | 84.4 | 132.4 | 98.4 | 132.4 | 42.7 |
| 195 | 18YQBF | 972.6 | 1013.6 | 972.6 | 697.6 | 972.6 | 882 | 972.6 | 467 |
| 195 | 0R479N | 509.1 | 495.1 | 509.1 | 2488.7 | 509.1 | 405.8 | 509.1 | 1931.9 |
| 195 | 0W708Z | 37.1 | 36.7 | 37.1 | 123.3 | 37.1 | 21.8 | 37.1 | 70.7 |
| 195 | 8A6KBK | 40.5 | 31.9 | 40.5 | 91.4 | 40.5 | 23.3 | 40.5 | 64.9 |
| 195 | 92HTUM | 4.8 | 3.5 | 4.8 | 12.4 | 4.8 | 3.2 | 4.8 | 7.5 |
| 195 | 49ZYT6 | 525.2 | 552.8 | 525.2 | 304.5 | 525.2 | 484.5 | 525.2 | 254.2 |
| 195 | 30A6BG | 662.8 | 739.7 | 662.8 | 509.2 | 662.8 | 553.4 | 662.8 | 326.4 |
| 195 | L9FPHH | 616 | 453 | 616 | 248.7 | 616 | 444 | 616 | 210.9 |
| 195 | QP7RRL | 238.9 | 181.5 | 238.9 | 99.2 | 238.9 | 169.9 | 238.9 | 77 |
| 195 | J2LHWB | 645.5 | 645.9 | 645.5 | 331.8 | 645.5 | 557.9 | 645.5 | 283.4 |
| 195 | CW3FX9 | 51.7 | 222.2 | 51.7 | 65.6 | 51.7 | 64.9 | 51.7 | 33.1 |
| 195 | I8TN6N | 13.6 | 9 | 13.6 | 7.4 | 13.6 | 7.4 | 13.6 | 5.7 |
| 196 | 14YEDZ | 7.6 | 2.8 | 7.6 | 1.5 | 7.6 | 4.2 | 7.6 | 0.6 |
| 196 | AFZN0R | 41.5 | 16.9 | 41.5 | 9.1 | 41.5 | 22.8 | 41.5 | 4.3 |
| 196 | BU7NMG | 14.4 | 5.2 | 14.4 | 3.5 | 14.4 | 8.2 | 14.4 | 2.8 |
| 196 | JKC32H | 42 | 21.4 | 42 | 13 | 42 | 22.2 | 42 | 7.2 |
| 196 | QXM1B7 | 216 | 63.5 | 216 | 26.1 | 216 | 132.2 | 216 | 27.1 |
| 196 | YZ06AV | 197.6 | 61.4 | 197.6 | 50.6 | 197.6 | 138.4 | 197.6 | 52.8 |
| 196 | BTM1YE | 648.8 | 462 | 648.8 | 350.3 | 648.8 | 524.8 | 648.8 | 263.3 |
| 196 | NKTC04 | 340.3 | 237.7 | 340.3 | 168.1 | 340.3 | 271.6 | 340.3 | 116.2 |
| 196 | 9BXG3M | 911.3 | 921 | 911.3 | 595.7 | 911.3 | 685.1 | 911.3 | 386.8 |
| 196 | AAY07D | 139.5 | 128.9 | 139.5 | 67.6 | 139.5 | 82 | 139.5 | 37.2 |
| 196 | EB48YM | 51.1 | 39.5 | 51.1 | 17.8 | 51.1 | 26.8 | 51.1 | 8.6 |
| 196 | 66SDN0 | 212.7 | 170.8 | 212.7 | 77.3 | 212.7 | 161.4 | 212.7 | 61.9 |
| 196 | 86F2TK | 64 | 48.4 | 64 | 23.2 | 64 | 47.2 | 64 | 21.3 |
| 196 | 4KW7H9 | 233.9 | 161.4 | 233.9 | 88.6 | 233.9 | 181.1 | 233.9 | 84 |
| 196 | JVEI5Y | 234.4 | 168.4 | 234.4 | 84 | 234.4 | 169.4 | 234.4 | 77.8 |
| 196 | N8DZQ8 | 84.6 | 22.7 | 84.6 | 6.9 | 84.6 | 29.8 | 84.6 | 5.8 |
| 196 | RLTX4G | 73.5 | 23.9 | 73.5 | 5.6 | 73.5 | 32.5 | 73.5 | 4 |
| 196 | YZB02J | 179.9 | 214 | 179.9 | 92.6 | 179.9 | 172 | 179.9 | 87.1 |
| 196 | 6DQ39T | 452.8 | 282.9 | 452.8 | 142.1 | 452.8 | 390.5 | 452.8 | 155.3 |
| 196 | CVJ130 | 314.9 | 235.9 | 314.9 | 123.4 | 314.9 | 273.7 | 314.9 | 117 |
| 196 | 2C7VNA | 270 | 388.5 | 270 | 156.7 | 270 | 235.9 | 270 | 101.8 |
| 196 | 63GP52 | 308.6 | 406.9 | 308.6 | 181 | 308.6 | 258.1 | 308.6 | 120.2 |
| 196 | 8LQR2U | 140.6 | 114.2 | 140.6 | 43.4 | 140.6 | 103.3 | 140.6 | 38.2 |
| 196 | J9AJ01 | 179.6 | 155.9 | 179.6 | 64.5 | 179.6 | 136.5 | 179.6 | 57.2 |

Data presents the mean value of three experimental replicate, each with three plants. 195 and 196 represent photosynthesis pathway (database KEGG map id 00195) and photosynthesis antenna pathway (database KEGG map id 00196), respectively.

Table S 3 The FPKM of differentially expressed genes in starch and sucrose metabolism

| Gene id | CK_CS40 | CS40 | CK_CS80 | CS80 | CK_S40 | FS40 | CK_FS80 | FS80 |
| --- | --- | --- | --- | --- | --- | --- | --- | --- |
| 0FY2NM | 0.68 | 0.16 | 0.68 | 0.00 | 0.68 | 0.05 | 0.68 | 0.00 |
| 0INE4H | 3.21 | 1.67 | 3.21 | 0.61 | 3.21 | 2.38 | 3.21 | 1.19 |
| 0Q5BJB | 8.32 | 3.62 | 8.32 | 1.64 | 8.32 | 3.97 | 8.32 | 0.78 |
| 0XM443 | 0.87 | 1.72 | 0.87 | 8.31 | 0.87 | 1.98 | 0.87 | 4.46 |
| 11MF2K | 0.98 | 1.16 | 0.98 | 0.22 | 0.98 | 1.04 | 0.98 | 0.20 |
| 1FB51V | 19.28 | 11.04 | 19.28 | 4.41 | 19.28 | 17.42 | 19.28 | 6.01 |
| 23AQDA | 24.30 | 19.00 | 24.30 | 9.58 | 24.30 | 18.53 | 24.30 | 7.27 |
| 2A3E3X | 0.50 | 0.23 | 0.50 | 0.00 | 0.50 | 1.05 | 0.50 | 0.10 |
| 2SN1MA | 31.66 | 32.77 | 31.66 | 4.22 | 31.66 | 35.68 | 31.66 | 8.15 |
| 31H9ZG | 0.71 | 2.09 | 0.71 | 0.82 | 0.71 | 2.40 | 0.71 | 1.73 |
| 40FSM2 | 0.39 | 0.31 | 0.39 | 0.96 | 0.39 | 0.81 | 0.39 | 1.29 |
| 41CPL5 | 0.14 | 0.57 | 0.14 | 1.27 | 0.14 | 0.43 | 0.14 | 0.59 |
| 477F6P | 0.00 | 0.05 | 0.00 | 0.39 | 0.00 | 0.05 | 0.00 | 0.11 |
| 489DAS | 1.08 | 0.65 | 1.08 | 0.19 | 1.08 | 0.93 | 1.08 | 0.38 |
| 4HU4LR | 23.79 | 17.34 | 23.79 | 9.49 | 23.79 | 20.34 | 23.79 | 21.71 |
| 4MRL1E | 62.59 | 33.81 | 62.59 | 22.68 | 62.59 | 48.35 | 62.59 | 19.90 |
| 4VZ14Z | 0.29 | 0.86 | 0.29 | 0.71 | 0.29 | 1.52 | 0.29 | 1.33 |
| 4Y2508 | 98.56 | 40.27 | 98.56 | 19.60 | 98.56 | 46.92 | 98.56 | 23.51 |
| 4Z6VDH | 4.28 | 0.66 | 4.28 | 0.16 | 4.28 | 1.38 | 4.28 | 0.13 |
| 53FINS | 59.60 | 26.30 | 59.60 | 4.13 | 59.60 | 39.07 | 59.60 | 7.19 |
| 5E5ZG3 | 1.07 | 1.45 | 1.07 | 3.08 | 1.07 | 2.85 | 1.07 | 6.21 |
| 5SXX56 | 20.64 | 22.41 | 20.64 | 4.95 | 20.64 | 27.36 | 20.64 | 8.70 |
| 5W32PJ | 10.94 | 7.97 | 10.94 | 4.13 | 10.94 | 7.74 | 10.94 | 4.85 |
| 6Y4DBB | 3.53 | 0.59 | 3.53 | 0.21 | 3.53 | 3.11 | 3.53 | 0.90 |
| 733TCI | 9.25 | 6.91 | 9.25 | 3.23 | 9.25 | 6.20 | 9.25 | 3.98 |
| 7NA3QC | 23.51 | 15.81 | 23.51 | 8.79 | 23.51 | 19.25 | 23.51 | 8.26 |
| 7Q0HRU | 26.24 | 22.61 | 26.24 | 5.46 | 26.24 | 26.57 | 26.24 | 9.75 |
| 7RJD9X | 0.78 | 0.95 | 0.78 | 9.22 | 0.78 | 1.79 | 0.78 | 4.30 |
| 7VB1JU | 25.42 | 21.89 | 25.42 | 11.65 | 25.42 | 22.83 | 25.42 | 33.68 |
| 86IGSK | 4.92 | 8.50 | 4.92 | 6.31 | 4.92 | 7.25 | 4.92 | 10.22 |
| 8EBA9D | 36.85 | 28.15 | 36.85 | 24.84 | 36.85 | 21.13 | 36.85 | 12.24 |
| 8UPE10 | 25.26 | 28.14 | 25.26 | 19.86 | 25.26 | 21.84 | 25.26 | 10.63 |
| B363MR | 35.12 | 28.30 | 35.12 | 4.48 | 35.12 | 25.67 | 35.12 | 3.62 |
| BL0RCS | 6.86 | 6.03 | 6.86 | 2.40 | 6.86 | 7.07 | 6.86 | 5.61 |
| C1I278 | 2.77 | 1.93 | 2.77 | 0.97 | 2.77 | 1.94 | 2.77 | 0.24 |
| CM68RF | 4.59 | 4.58 | 4.59 | 8.13 | 4.59 | 6.04 | 4.59 | 9.50 |
| CZGL8I | 2.36 | 4.00 | 2.36 | 3.50 | 2.36 | 4.82 | 2.36 | 4.33 |
| D91BSU | 76.86 | 33.17 | 76.86 | 31.40 | 76.86 | 54.55 | 76.86 | 26.00 |
| E3NNBV | 13.25 | 10.12 | 13.25 | 4.18 | 13.25 | 11.54 | 13.25 | 5.51 |
| E48PAY | 3.71 | 3.54 | 3.71 | 1.12 | 3.71 | 3.40 | 3.71 | 1.44 |
| E80PU7 | 0.53 | 0.73 | 0.53 | 1.71 | 0.53 | 0.59 | 0.53 | 0.84 |
| EBDX7F | 91.52 | 60.64 | 91.52 | 22.00 | 91.52 | 66.74 | 91.52 | 26.76 |
| EC4LZV | 13.68 | 14.31 | 13.68 | 3.91 | 13.68 | 15.94 | 13.68 | 8.38 |
| EEUI5F | 12.96 | 7.21 | 12.96 | 0.52 | 12.96 | 8.13 | 12.96 | 0.80 |
| F91WU6 | 85.40 | 38.67 | 85.40 | 15.63 | 85.40 | 73.63 | 85.40 | 25.52 |
| FIK9JS | 1.87 | 3.48 | 1.87 | 0.78 | 1.87 | 2.42 | 1.87 | 1.65 |
| FP7P7G | 4.14 | 5.31 | 4.14 | 19.64 | 4.14 | 5.38 | 4.14 | 12.69 |
| G2WE73 | 0.87 | 0.15 | 0.87 | 0.04 | 0.87 | 0.11 | 0.87 | 0.01 |
| G79KWN | 54.08 | 25.34 | 54.08 | 18.72 | 54.08 | 43.95 | 54.08 | 21.06 |
| G9F7ZA | 22.96 | 10.91 | 22.96 | 8.30 | 22.96 | 24.25 | 22.96 | 8.89 |
| GJKP1U | 0.53 | 1.18 | 0.53 | 0.94 | 0.53 | 2.47 | 0.53 | 2.00 |
| HP9A0E | 1.87 | 2.56 | 1.87 | 0.78 | 1.87 | 2.37 | 1.87 | 0.86 |
| HZCB2X | 40.61 | 17.12 | 40.61 | 10.25 | 40.61 | 23.57 | 40.61 | 7.78 |
| IC3HGI | 4.34 | 3.48 | 4.34 | 1.31 | 4.34 | 4.86 | 4.34 | 3.88 |
| IE8W25 | 6.57 | 7.27 | 6.57 | 12.84 | 6.57 | 8.32 | 6.57 | 14.97 |
| IEIY3V | 0.73 | 0.12 | 0.73 | 0.04 | 0.73 | 0.26 | 0.73 | 0.00 |
| IXM19M | 2.92 | 0.98 | 2.92 | 0.48 | 2.92 | 1.36 | 2.92 | 0.23 |
| J31KW7 | 1.68 | 1.58 | 1.68 | 0.57 | 1.68 | 1.30 | 1.68 | 0.61 |
| J4P8M7 | 7.26 | 4.10 | 7.26 | 3.43 | 7.26 | 5.74 | 7.26 | 4.15 |
| JGCP84 | 2.00 | 0.93 | 2.00 | 0.71 | 2.00 | 1.37 | 2.00 | 0.52 |
| JMYD5Y | 16.56 | 13.31 | 16.56 | 6.12 | 16.56 | 14.87 | 16.56 | 8.56 |
| JTF09J | 4.41 | 2.39 | 4.41 | 0.56 | 4.41 | 3.15 | 4.41 | 1.07 |
| K29YM9 | 23.02 | 14.31 | 23.02 | 10.69 | 23.02 | 21.01 | 23.02 | 10.79 |
| KWM0H4 | 21.05 | 8.49 | 21.05 | 10.66 | 21.05 | 23.04 | 21.05 | 15.55 |
| KZA261 | 1.83 | 1.57 | 1.83 | 0.27 | 1.83 | 1.63 | 1.83 | 0.64 |
| L4CNUE | 0.61 | 1.25 | 0.61 | 1.37 | 0.61 | 3.01 | 0.61 | 4.74 |
| L6ZHES | 9.86 | 2.52 | 9.86 | 0.36 | 9.86 | 3.34 | 9.86 | 0.18 |
| LC98AP | 44.07 | 29.63 | 44.07 | 8.88 | 44.07 | 44.61 | 44.07 | 17.57 |
| M043T1 | 66.82 | 41.89 | 66.82 | 32.38 | 66.82 | 40.10 | 66.82 | 16.25 |
| M8RGP5 | 9.87 | 7.34 | 9.87 | 3.64 | 9.87 | 8.97 | 9.87 | 4.91 |
| M8S3TB | 53.22 | 11.75 | 53.22 | 2.12 | 53.22 | 18.95 | 53.22 | 2.67 |
| MS236J | 29.12 | 18.23 | 29.12 | 6.56 | 29.12 | 23.95 | 29.12 | 10.80 |
| MTP01V | 3.44 | 3.30 | 3.44 | 1.79 | 3.44 | 3.06 | 3.44 | 1.30 |
| N1N6FA | 11.64 | 14.82 | 11.64 | 4.26 | 11.64 | 15.71 | 11.64 | 9.35 |
| NH7NMJ | 0.00 | 0.02 | 0.00 | 0.08 | 0.00 | 0.01 | 0.00 | 0.19 |
| NTRM8G | 0.47 | 1.02 | 0.47 | 1.36 | 0.47 | 0.79 | 0.47 | 0.90 |
| P5P8C7 | 0.07 | 0.51 | 0.07 | 0.00 | 0.07 | 0.21 | 0.07 | 0.09 |
| PFNU0X | 43.90 | 19.34 | 43.90 | 9.98 | 43.90 | 27.45 | 43.90 | 10.87 |
| PH1SAB | 0.16 | 0.03 | 0.16 | 0.07 | 0.16 | 0.06 | 0.16 | 0.00 |
| PT6J7Z | 0.66 | 1.20 | 0.66 | 3.33 | 0.66 | 0.46 | 0.66 | 2.07 |
| PX7TTT | 2.92 | 7.77 | 2.92 | 17.72 | 2.92 | 6.68 | 2.92 | 12.44 |
| Q3GMD8 | 2.47 | 3.63 | 2.47 | 15.50 | 2.47 | 3.82 | 2.47 | 11.88 |
| QR17ZP | 2.12 | 1.77 | 2.12 | 0.47 | 2.12 | 3.00 | 2.12 | 1.81 |
| QX73S9 | 0.51 | 0.52 | 0.51 | 0.22 | 0.51 | 0.73 | 0.51 | 0.05 |
| R34KW1 | 0.68 | 0.24 | 0.68 | 0.05 | 0.68 | 0.19 | 0.68 | 0.04 |
| R8PHGQ | 22.15 | 9.49 | 22.15 | 4.91 | 22.15 | 16.64 | 22.15 | 6.59 |
| RCXU7Z | 0.45 | 0.86 | 0.45 | 1.04 | 0.45 | 0.90 | 0.45 | 1.72 |
| SB83T1 | 0.92 | 2.32 | 0.92 | 1.50 | 0.92 | 2.11 | 0.92 | 2.47 |
| SD1YX7 | 95.66 | 53.64 | 95.66 | 20.61 | 95.66 | 69.24 | 95.66 | 28.47 |
| SE6XPK | 3.28 | 3.39 | 3.28 | 0.73 | 3.28 | 4.25 | 3.28 | 2.14 |
| T6QQUE | 11.43 | 10.10 | 11.43 | 5.41 | 11.43 | 12.24 | 11.43 | 6.19 |
| TB97SW | 1.37 | 1.84 | 1.37 | 0.35 | 1.37 | 1.97 | 1.37 | 1.24 |
| THXS6I | 2.66 | 2.24 | 2.66 | 0.82 | 2.66 | 1.75 | 2.66 | 1.18 |
| TP5BL7 | 130.76 | 70.07 | 130.76 | 28.08 | 130.76 | 76.94 | 130.76 | 34.19 |
| UEEX8A | 18.88 | 6.37 | 18.88 | 1.82 | 18.88 | 13.18 | 18.88 | 2.05 |
| UW0ZL7 | 8.43 | 8.18 | 8.43 | 3.32 | 8.43 | 9.30 | 8.43 | 5.35 |
| V374FJ | 19.19 | 5.19 | 19.19 | 1.22 | 19.19 | 7.03 | 19.19 | 0.94 |
| V3P7C9 | 1.39 | 0.97 | 1.39 | 0.55 | 1.39 | 1.43 | 1.39 | 1.09 |
| VAT0VP | 21.23 | 8.02 | 21.23 | 7.22 | 21.23 | 18.36 | 21.23 | 8.57 |
| VE7NYG | 26.49 | 20.69 | 26.49 | 13.92 | 26.49 | 15.10 | 26.49 | 8.84 |
| W2MXXV | 0.25 | 0.10 | 0.25 | 0.02 | 0.25 | 0.05 | 0.25 | 0.00 |
| W2WG0I | 2.98 | 0.93 | 2.98 | 0.79 | 2.98 | 2.11 | 2.98 | 0.62 |
| WCV94E | 125.56 | 69.34 | 125.56 | 47.27 | 125.56 | 94.35 | 125.56 | 32.51 |
| WF73FH | 3.07 | 2.91 | 3.07 | 1.75 | 3.07 | 2.97 | 3.07 | 1.02 |
| WZ5PI0 | 0.06 | 0.09 | 0.06 | 0.37 | 0.06 | 0.12 | 0.06 | 0.27 |
| XSZ7IL | 1.93 | 1.30 | 1.93 | 0.70 | 1.93 | 1.84 | 1.93 | 1.24 |
| Y7IGXD | 1.17 | 0.36 | 1.17 | 0.24 | 1.17 | 0.76 | 1.17 | 0.01 |
| Y7NYXA | 0.12 | 0.25 | 0.12 | 0.67 | 0.12 | 0.18 | 0.12 | 0.63 |
| YDQ82R | 10.97 | 4.68 | 10.97 | 2.61 | 10.97 | 5.35 | 10.97 | 2.86 |
| YJI1GZ | 3.24 | 3.66 | 3.24 | 0.99 | 3.24 | 4.38 | 3.24 | 2.80 |
| YT3TY8 | 2.47 | 4.13 | 2.47 | 3.63 | 2.47 | 4.13 | 2.47 | 7.10 |
| YY1VWG | 7.74 | 7.52 | 7.74 | 2.86 | 7.74 | 7.03 | 7.74 | 3.46 |
| ZRSS3A | 1.37 | 0.58 | 1.37 | 0.14 | 1.37 | 3.21 | 1.37 | 0.68 |

Data presents the mean value of three experimental replicate, each with three plants. The sample name with prefix CK represents the control for the corresponding shade treated sample.

Table S4 RNA-Seq data accession, sample name and treatment at NCBI

| Data files | NCBI Accession # | Samples | Treatment |
| --- | --- | --- | --- |
| B4030-1_BKDL190834182-1a_1  B4030-1_BKDL190834182-1a_2 | SRR11659555 | S4030-1 | FS40  40% shade  for 30 days  flowering stage |
| B4030-2_BKDL190834183-1a_1  B4030-2_BKDL190834183-1a_2 | SRR11659545 | S4030-2 |  |
| B4030-3_BKDL190834184-1a_1  B4030-3_BKDL190834184-1a_2 | SRR11659544 | S4030-3 |  |
| B8030-1_BKDL190834185-1a_1  B8030-1_BKDL190834185-1a_2 | SRR11659543 | S8030-1 | FS80  80% shade  for 30 days  flowering stage |
| B8030-2_BKDL190834186-1a_1  B8030-2_BKDL190834186-1a_2 | SRR11659538 | S8030-2 |  |
| B8030-3_BKDL190834187-1a_1  B8030-3_BKDL190834187-1a_2 | SRR11659537 | S8030-3 |  |
| C40-1_BKDL190834188-1a_1  C40-1_BKDL190834188-1a_2 | SRR11659536 | CS40-1 | CS40  40% shade  for 60 days  Seedling +  flowering stage |
| C40-2_BKDL190834189-1a_1  C40-2_BKDL190834189-1a_2 | SRR11659540 | CS40-2 |  |
| C40-3_BKDL190834190-1a_1  C40-3_BKDL190834190-1a_2 | SRR11659539 | CS40-3 |  |
| C80-1_BKDL190834191-1a_1  C80-1_BKDL190834191-1a_2 | SRR11659574 | CS80-1 | CS80  80% shade  for 60 days  Seedling +  flowering stage |
| C80-2_BKDL190834192-1a_1  C80-2_BKDL190834192-1a_2 | SRR11659573 | CS80-2 |  |
| C80-3_BKDL190834193-1a_1  C80-3_BKDL190834193-1a_2 | SRR11659539 | CS80-3 |  |
| CK-1_BKDL190834179-1a_1  CK-1_BKDL190834179-1a_2 | SRR11659542 | CK-1 | CK  (Control) |
| CK-2_BKDL190834180-1a_1  CK-2_BKDL190834180-1a_2 | SRR11659541 | CK-2 |  |
| CK-3_BKDL190834181-1a_1  CK-3_BKDL190834181-1a_2 | SRR11659566 | CK-3 |  |
